# Biosynthesis of the modified tetrapyrroles—the pigments of life

**DOI:** 10.1074/jbc.REV120.006194

**Published:** 2020-04-02

**Authors:** Donald A. Bryant, C. Neil Hunter, Martin J. Warren

**Affiliations:** ‡ Department of Biochemistry and Molecular Biology, The Pennsylvania State University, University Park, Pennsylvania 16802; § Department of Chemistry and Biochemistry, Montana State University, Bozeman, Montana 59717; ¶ Department of Molecular Biology and Biotechnology, University of Sheffield, Sheffield S10 2TN, United Kingdom; ‖ School of Biosciences, University of Kent, Canterbury CT2 7NJ, United Kingdom; ** Quadram Institute Bioscience, Norwich Research Park, Norwich NR4 7UQ, United Kingdom

**Keywords:** heme, chlorophyll, biosynthesis, adenosylcobalamin (AdoCbl), photosynthesis, bacteriochlorophyll, bilin, tetrapyrrole, uroporphyrinogen III, vitamin B12, cobalamin, coenzyme F430, heme d1, 5-aminolevulinic acid, precorrin

## Abstract

Modified tetrapyrroles are large macrocyclic compounds, consisting of diverse conjugation and metal chelation systems and imparting an array of colors to the biological structures that contain them. Tetrapyrroles represent some of the most complex small molecules synthesized by cells and are involved in many essential processes that are fundamental to life on Earth, including photosynthesis, respiration, and catalysis. These molecules are all derived from a common template through a series of enzyme-mediated transformations that alter the oxidation state of the macrocycle and also modify its size, its side-chain composition, and the nature of the centrally chelated metal ion. The different modified tetrapyrroles include chlorophylls, hemes, siroheme, corrins (including vitamin B_12_), coenzyme F_430_, heme *d*_1_, and bilins. After nearly a century of study, almost all of the more than 90 different enzymes that synthesize this family of compounds are now known, and expression of reconstructed operons in heterologous hosts has confirmed that most pathways are complete. Aside from the highly diverse nature of the chemical reactions catalyzed, an interesting aspect of comparative biochemistry is to see how different enzymes and even entire pathways have evolved to perform alternative chemical reactions to produce the same end products in the presence and absence of oxygen. Although there is still much to learn, our current understanding of tetrapyrrole biogenesis represents a remarkable biochemical milestone that is summarized in this review.

## Introduction

**Figure 1.**
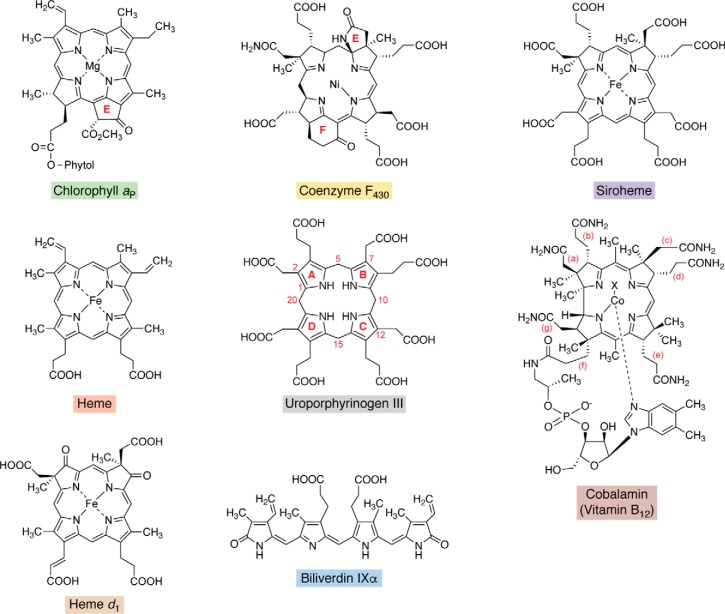
**Structures of the major modified tetrapyrroles outlined in this review and their structural relationship to the first macrocyclic primogenitor, uroporphyrinogen III.** The major modified tetrapyrroles shown surrounding the central uroporphyrinogen III include chlorophyll *a*_P_, coenzyme F_430_, siroheme, cobalamin, biliverdin IXα, heme *d*_1_, and heme *b*. The asymmetrically arranged pyrrole rings in uroporphyrinogen III are named A–D, with the D ring inverted with respect to the other rings. The numbering scheme for the macrocycle is shown for uroporphyrinogen, where positions 1, 2, 5, 7, 10, 12, 15, and 20 are highlighted. In Chls there is a fifth ring, termed ring E, and similarly in F_430_, there are two extra rings that are termed E and F as shown. For cobalamin (vitamin B_12_), the side chains are designated (*a–f*), and these are *labeled*. The *X above* the cobalt is a cyanide group in vitamin B_12_; this position is occupied by either a methyl or adenosyl group in the major biological forms of cobalamin. The *shaded boxes* surrounding the names of these end-product compounds coordinate with the *colors* in other pathway figures and in the summary pathway depicted in Fig. 14.

Modified tetrapyrroles play essential roles in a broad range of essential biological processes. Their large macrocyclic structures and diverse conjugation and metal chelation systems also provide an array of colors, such that they have been dubbed the “pigments of life” (1). These life pigments include the hemes, chlorophylls (Chls),^4^ bilins, corrins (vitamin B_12_), siroheme, and coenzyme F_430_. They are all made from a single, extensively branched biosynthetic pathway and are based on the blueprint of a common biosynthetic primogenitor, uroporphyrinogen III (Fig. 1). These different modified tetrapyrroles vary in the nature of their peripheral side chains, the oxidation state of the macrocycle itself, and the centrally chelated metal ion. Perhaps the most distinctive of all is vitamin B_12_, which contains a ring-contracted macrocycle and also houses upper and lower ligands in order to provide the octahedral geometry to coordinate the cobalt ion.

The differences in the structures of these molecules are reflected in diverse biological functions. Some of the modified tetrapyrroles are involved in very specific processes; for example, heme *d*_1_ is required as a prosthetic group only for the *cd*_1_ nitrite reductase (2), whereas others, such as heme, are involved in a myriad of distinct biological roles, from sensing to catalysis (3). A brief description of the roles played by these molecules is outlined below.

Chls and the related bacteriochlorophylls (BChls) are the molecules that not only give plants their green pigmentation but are intricately involved in the process of photosynthesis (4). Chls play two roles in photosynthesis: first, they act as antenna molecules and harvest solar energy, and second, they transfer this energy to the reaction centers, where photochemistry occurs that results in the splitting of water or the production of strong reductants for carbon dioxide fixation and ATP generation. With only a few exceptions in which Zn^2+^ replaces Mg^2+^, Chls are Mg^2+^-containing chlorins, and the electronic properties of the chlorin ring allow for the efficient formation of a singlet excited state upon visible light absorption. In contrast to the metal ions found in other modified tetrapyrroles, Mg^2+^ is not redox-active, and the metal does not play a direct role in the light-trapping process. However, the metal does appear to help potentiate the chemistry of the chlorin ring to make energy transfer more efficient.

Heme is technically an Fe-containing porphyrin. It has one more double bond in the macrocycle than Chls, and the extra conjugation helps produce the red color associated with the molecule. The central Fe ion is crucial to the functions for which heme is used (5). Iron exists in several oxidation states, and, for this reason, heme has evolved a broad range of roles within biological systems, from acting as a one-electron carrier in respiratory cytochromes to a sensing role for a range of diatomic gases, including CO, NO, and O_2_. Heme also acts as the prosthetic group in a range of enzymes, including catalases, peroxidases, and cytochromes P_450_, and is known to be associated with certain transporters and transcription factors (3).

The corrinoids, sometimes also referred to as cobamides, encompass cofactors and coenzymes that harbor cobalt-containing, ring-contracted corrin macrocycles. In biologically active corrinoids, the cobalt atom is generally found covalently linked to either a methyl or adenosyl group on the upper face of the macrocycle. The corrin ring is also attached to a lower nucleotide loop via one of its propionate side chains. The nature of the base in this nucleotide loop varies among bacteria, and over 20 different bases are known to be incorporated into corrinoids. The base is specifically dimethylbenzimidazole in vitamin B_12_, and this specific corrinoid appears to be the only form utilized by eukaryotes. Corrinoids appear to be involved in an ever-increasing number of roles (6). Methylcorrinoids act as the coenzyme in methyl transfer reactions, such as those mediated by methionine synthase (7). Adenosylcorrinoids act as the coenzyme in a number of different rearrangement reactions that are mediated by an adenosyl radical, formed from the homolytic cleavage of the Co-adenosyl bond. These reactions include, among many others, methylmalonyl-CoA mutase, ribonucleotide reductase, and the diol dehydratases (7). Adenosylcobalamin has also recently been shown to be involved as a light sensor in a transcription factor (8). Corrinoids without an upper ligand act as the catalytic center for reductive dehalogenases, in which the cobalt ion is thought to form a direct bond with the halide component of the substrate in order to mediate its abstraction (9). Finally, both methylcorrinoids and adenosylcorrinoids also appear to be involved in a specific group of radical SAM enzymes (6); B_12_-radical SAM enzymes are the largest group within this broad enzyme class (10).

Coenzyme F_430_ is a nickel-containing tetrahydroporphyrinogen and acts as a coenzyme in both forward and reverse methanogenesis (11). As a coenzyme within coenzyme M reductase, the central nickel ion is able to mediate the reversible reduction/oxidation of a methyl group to produce methane in both the processes of methanogenesis and anaerobic methane oxidation. In some respects, the binding of nickel in coenzyme F_430_ mirrors the binding of cobalt in corrins, reflecting similarities in their respective catalytic activities in forming metal-carbon bonds. However, in coenzyme F_430_, nickel promotes methyl group reduction, whereas in corrinoid-dependent methyltransferases, the cobalt promotes methyl group transfer (12). Demonstrating the importance of these tetrapyrrole catalysts, the process of methanogenesis is responsible for the overall production of around 1 billion tons of methane gas per year (12).

The final two modified cyclic tetrapyrroles are siroheme and heme *d*_1_. Siroheme is the simplest of the modified tetrapyrroles, and is an Fe-containing isobacteriochlorin (13). It is found mainly in sulfite reductases but also some assimilatory nitrite reductases. The prosthetic group assists in the 6-electron reduction of both sulfite and nitrite to allow their incorporation into biological systems at the level of sulfide and ammonia. It has been suggested that siroheme, in preference to heme, allows a more direct charge transfer route to the active center of these enzymes during the catalytic cycle (14). In contrast, heme *d*_1_, which like siroheme is not a heme but a dioxo-isobacteriochlorin, is only utilized by one enzyme, a dissimilatory nitrite reductase called cytochrome *cd*_1_.

Most bilins are derived from heme by oxidative cleavage of the macrocycle to produce biliverdin with release of CO (15, 16). Ferredoxin-dependent bilin reductases as well as isomerases then lead to the production of four principal bilin pigments: phycocyanobilin, phycoerythrobilin, phycoviolobilin, and phycourobilin. Bilins form the chromophores of phycobiliproteins (17, 18) or the light-sensing photosensors of sensor histidine kinases, including phytochromes or cyanobacteriochromes (19, 20).

As stated earlier, all of these modified tetrapyrroles are synthesized along a branched biochemical pathway from the first and only common macrocyclic intermediate, uroporphyrinogen III. In this review, we will deal with the biogenesis of uroporphyrinogen III and then detail how this macrocycle is converted into the various metallo-prosthetic groups that make up this unique but ubiquitous family of essential life pigments.

## Biosynthesis of uroporphyrinogen III

**Figure 2.**
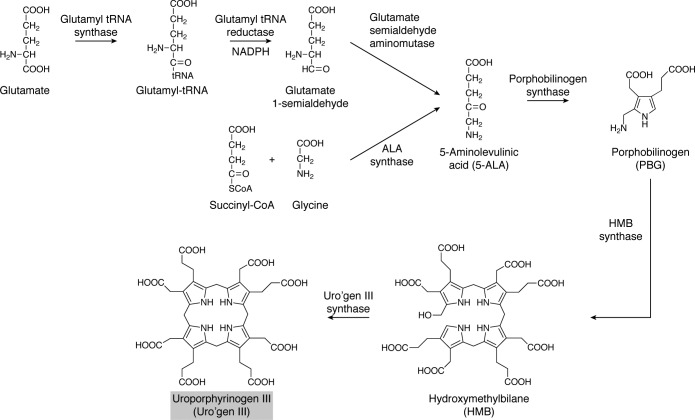
**The two routes for the biosynthesis of 5-ALA and the subsequent biosynthesis of uroporphyrinogen III.** The Shemin, or C4, route involves the condensation of glycine and succinyl-CoA and is mediated by the enzyme 5-aminolevulinic acid synthase. The C5 pathway acquires the intact carbon skeleton from glutamate and utilizes glutamyl-tRNA as an intermediate. The glutamyl-tRNA undergoes a reduction by glutamyl-tRNA reductase to give GSA. The final step involves the enzyme GsaM, which rearranges the GSA into 5-ALA. Then two molecules of 5-ALA are condensed into PBG by the action of the enzyme porphobilinogen synthase. Next, four molecules of PBG are deaminated and linked together to give a linear bilane called HMB in a reaction catalyzed by HMB synthase. The final step involves the cyclization and inversion of the terminal D ring to give uroporphyrinogen III. The *gray box* for uroporphyrinogen III also identifies this central intermediate in Figs. 3 and 14.

The common precursor metabolite for the synthesis of uroporphyrinogen III is the amino ketone, 5-aminolevulinic acid (5-ALA) (21). This C5 intermediate is uniquely used for the biosynthesis of modified tetrapyrroles and is made by one of two routes (Fig. 2). Some organisms make 5-ALA from a decarboxylating condensation between succinyl-CoA and glycine, called the C4 or Shemin pathway, whereas others make 5-ALA from the intact carbon skeleton of glutamate, called the C5 route. We will briefly review these two pathways and describe how 5-ALA is synthesized.

### The C4 pathway

The C4 or Shemin route was discovered by Shemin and Rittenberg (22), who had previously shown that the nitrogen atom from glycine was incorporated into heme through labeling studies. Subsequently, Shemin's laboratory and that of Albert Neuberger demonstrated independently that succinyl-CoA condensed with glycine to give 5-ALA (23, 24). The C4 route is mediated by a single enzyme called 5-aminolevulinic acid synthase (AlaS) (Fig. 2). This enzyme is found in some α-proteobacteria and most eukaryotic organisms apart from higher plants. As well as utilizing both succinyl-CoA and glycine as substrates, the enzyme also employs pyridoxal phosphate as a cofactor. The reaction proceeds through the binding of glycine to the pyridoxal phosphate, forming a Schiff base within the active site. Proton abstraction, followed by attachment of the succinyl-CoA, generates 2-amino-3-ketoadipate as a transient intermediate. Loss of CoA and CO_2_ then leads to release of 5-ALA from the enzyme. The structure of AlaS from *Rhodobacter capsulatus* was the first to be solved, confirming that the enzyme is a homodimer (25). The structure provided many molecular details concerning the active-site residues and their roles in catalysis.

### The C5 pathway

The detection of AlaS in mammalian and some bacterial systems led to a hunt for the enzyme in other organisms and higher plants. However, no activity could be found, and it took some years before labeling studies with glutamate revealed that plants make 5-ALA from glutamate (26). It was subsequently shown, through a number of elegant experiments, that glutamate is converted into glutamate semialdehyde (GSA) and then into 5-ALA (27). The conversion of glutamate into GSA was shown to involve several steps. In fact, it is not glutamate itself that is converted into GSA, but rather glutamyl-tRNA, the same species that is used for mRNA translation. The involvement of glutamyl-tRNA represents one of the few examples in which a tRNA species is used in a process other than protein synthesis.

The enzyme that catalyzes the transformation of glutamyl-tRNA into GSA is called glutamyl-tRNA reductase, which is now known as GtrR. The enzyme requires NADPH and mediates the synthesis of the aldehyde. The structure of the *Methanopyrus kandleri* enzyme reveals that the homodimeric protein forms a large asymmetric V-shaped molecule, with a number of distinct domains located along a large helix (28). An active-site cysteine residue in the catalytic domain attacks the glutamate-tRNA bond to form an enzyme thioester with the release of the tRNA moiety. Reduction of the thioester bond by NADPH generates GSA (29). The shape of the enzyme permits an interaction with the next enzyme in the pathway, the GSA aminomutase, GsaM (30). The interaction between GtrR and GsaM allows for channeling of the relatively unstable GSA from one active site to the next. GsaM is also a member of the aminotransferase family and is similar to AlaS (31). The protein utilizes pyridoxamine-5′-phosphate as a cofactor to facilitate the rearrangement of the amino group to the C5 position of the molecule. The GSA binds to the pyridoxamine to generate 5′-diaminovalerate, which after rearrangement results in the formation of 5-ALA (32).

## Conversion of 5-ALA into uroporphyrinogen III

The transformation of 5-ALA into uroporphyrinogen III involves the actions of three enzymes (Fig. 2). These steps are common to all organisms that make modified tetrapyrroles, and no alternative route for the synthesis of uroporphyrinogen III has been described. Initially, 5-ALA is acted upon by an enzyme commonly called porphobilinogen synthase (PbgS), but it is also known as ALA dehydratase. The enzyme oversees a Knorr-type condensation reaction, in which two molecules of ALA are condensed to give the pyrrole porphobilinogen (PBG) (33). A significant amount of mechanistic work has shown that the first ALA molecule to bind to the enzyme gives rise to the propionate side chain of the product and that the second incoming molecule is incorporated into the acetic acid side of the molecule (34). The structures of a number of PbgS enzymes have been determined by protein crystallization and X-ray diffraction studies. The yeast enzyme was the first to be determined, and the structure revealed that the enzyme exists as a homooctamer (35). Two important lysine residues occur at the active site of the enzyme and bind the two incoming 5-ALA molecules to form Schiff bases (33). The yeast enzyme contains two Zn^2+^ ions; one of these is catalytically active, whereas the second appears to play a structural role. In other systems, Mg^2+^ plays a catalytic role, suggesting that the enzymes utilize the metals to act as Lewis acids within the reaction (36).

Four molecules of PBG are next polymerized into a linear bilane (Fig. 2), called hydroxymethylbilane (HMB), which involves the deamination of each of the substrates prior to their incorporation into the product in a highly ordered fashion (21). The enzyme is known as either HMB synthase (HmbS) or PBG deaminase. It is a monomeric enzyme with a molecular mass of around 35 kDa. It was the first enzyme involved in tetrapyrrole biosynthesis to be crystallized and to have its structure determined (37). The enzyme contains a very unusual cofactor, a dipyrromethane cofactor, which is also constructed from PBG (38, 39) and which is unique to tetrapyrrole biosynthesis. The cofactor is attached to cysteine 242 of the *Escherichia coli* enzyme through a thioether linkage (40). The free α position of the cofactor acts as the elongation site for HMB synthesis (41). The first PBG unit enters the active site and is deaminated through the actions of a catalytic aspartic acid residue. The resulting azafulvene then reacts with the dipyrromethane cofactor, in essence to form a tripyrrole. This first binding pyrrole unit ends up as ring A in the final tetrapyrrole macrocycle. This reaction sequence of PBG binding at the active site, deamination, and attachment to the free α position of the growing polypyrrole chain is repeated three more times until a hexapyrrole is formed, adding in rings B, C, and D of the final macrocycle. At this a point, the link between ring A and the dipyrromethane cofactor is hydrolyzed to generate HMB. The structure of the enzyme reveals a flexible active-site cavity that is lined with a number of arginine residues that help to stabilize and hold the growing polypyrrole entity (37).

The final step in the synthesis of uroporphyrinogen III is catalyzed by uroporphyrinogen III synthase (UroS) (Fig. 2). This enzyme not only cyclizes the HMB substrate but also inverts ring D of the bilane (21). In this respect, the first three rings of the uroporphyrinogen III product are arranged with their acetic acid and propionic acid side chains in the same order, whereas this order is reversed for ring D. This generates the only asymmetric isomer of uroporphyrinogen, providing a molecular handle in terms of substrate orientation with regard to future enzymatic steps (21). Uroporphyrinogen III synthase is a relatively small, monomeric enzyme with a molecular mass of around 25 kDa. A number of different enzyme mechanisms have been proposed for the enzyme to explain how it is able to mediate ring closure and rearrangement. The only one of these mechanisms to stand up to scrutiny is the spiro mechanism, which involves the formation of a cyclic spiro intermediate, allowing ring D to flip over. A chemically synthesized spiro-lactam analog of this intermediate was shown to act as a strong competitive inhibitor (42). This evidence suggests that the reaction does indeed proceed via a spiro intermediate en route to the formation of uroporphyrinogen III. Surprisingly, for what seems like a complicated reaction, there is very little alignment between the amino acid sequences of uroporphyrinogen III synthases, and no essential active site residue has been identified. However, a number of structures for the enzymes have been elucidated, including one with a bound product (43). Mutagenesis studies have identified a possible active-site tyrosine residue that may be involved in the elimination of the hydroxyl group from HMB (44).

**Figure 3.**
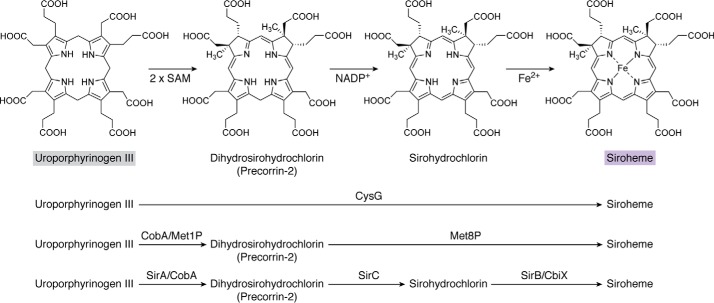
**The biosynthesis of siroheme from uroporphyrinogen III.** Initially, uroporphyrinogen is methylated at positions C2 and C7 to give precorrin-2 and then undergoes dehydrogenation to give sirohydrochlorin and finally ferrochelation to yield siroheme. The reactions are either mediated by three independent enzymes, such as SirA, -C, and -B, or by two enzymes, such as a uroporphyrinogen methyltransferase (CobA or Met1p)) and a bifunctional dehydrogenase/chelatase (Met8p), or by a single multifunctional enzyme, CysG. The *shaded boxes* surrounding the names of compounds coordinate with other pathway figures and the summary depiction in Fig. 14.

The biosynthesis of uroporphyrinogen III represents the first major branch point in tetrapyrrole biosynthesis (Fig. 3). Methylation of uroporphyrinogen III at positions 2 and 7 gives rise to precorrin-2, a highly unstable dipyrrocorphin that directs metabolism toward siroheme, cobalamin, and coenzyme F_430_ biogenesis. In contrast, decarboxylation of uroporphyrinogen III directs the intermediate toward protoporphyrin IXα (ProtoIX) and the biosynthesis of heme and Chls (21). These two branches will be considered separately, although, as will be seen later, heme can made from either ProtoIX or precorrin-2.

## The precorrin-2 branch

### The biosynthesis of siroheme

Siroheme represents the simplest of the modified tetrapyrroles. It is synthesized in just three steps from uroporphyrinogen III (Fig. 3). Initially, uroporphyrinogen III is methylated at positions C2 and C7 of the macrocyclic ring by uroporphyrinogen III methyltransferase, which requires SAM as a methyl donor (45). It is thought that the C2 position is methylated first, followed by C7. The resulting product, precorrin-2 (46), is also an intermediate in the biosynthesis of vitamin B_12_, heme *d*_1_, and coenzyme F_430_. The term precorrin arises from its first identification as an intermediate in the biosynthesis of the corrin ring, and all intermediates up to the formation of the corrin ring were given the name precorrin-*n*, where *n* refers to the number of methyl groups that have been added to the macrocycle (47). Therefore, precorrin-1 would be the intermediate for which a methyl group has only been added to the C2 position.

A number of structures of uroporphyrinogen III methyltransferases have been determined, showing a bi-lobal, kidney-shaped enzyme with the active site found at the junction of the two domains (48). The presence of SAH, one of the products of the methyl transfer reaction, is often seen in the active site and helps to pinpoint where the tetrapyrrole substrate is likely to bind. Indeed, a structure of a uroporphyrinogen methyltransferase with uroporphyrinogen III bound has also been determined (49). A conserved arginine residue may play a role in helping to promote the methylation events. It is assumed that uroporphyrinogen III binds in the correct orientation to allow methylation at the C2 position and that it then disengages, along with SAH, from the enzyme. After reloading with SAM, the precorrin-1 rebinds with the C7 positioned in close proximity to the SAM to allow the second methylation to take place.

Precorrin-2 is next acted upon by an NAD^+^-dependent dehydrogenase, which removes two protons and two electrons from the macrocycle, thereby introducing an extra double bond. This forms an isobacteriochlorin that is called sirohydrochlorin (Fig. 3). In some bacteria, the dehydrogenase exists as a single enzyme called SirC (50). In other systems, the dehydrogenase also has chelatase activity. One such example of a bifunctional dehydrogenase and chelatase is Met8p from yeast (51). This enzyme appears to use the same active site for both the dehydrogenase and chelatase activities. For the chelatase reaction, the enzyme has to insert ferrous iron into sirohydrochlorin to produce siroheme. Although the structure of Met8p has been determined, it is not clear how the chelation reaction is catalyzed or what residues are responsible for the process.

The overall chelation reaction involves the removal of the two protons attached to the pyrrole nitrogens. In contrast to the lack of information available for the chelatase activity of Met8p, single-function chelatases are also known in different bacterial systems. This includes the SirB enzyme (52), which is related to the chelatases associated with cobalt insertion into vitamin B_12_ via the anaerobic route, ferrochelatases associated with heme synthesis, and the nickel chelatase of F_430_ synthesis (53). These chelatases are classified as type II chelatases and are generally single-subunit enzymes that do not require ATP for metal insertion (53). This contrasts to the type I chelatases associated with Mg^2+^ insertion during Chl synthesis and cobalt insertion along the aerobic B_12_ pathway. Structural detail on the type II chelatases has helped identify the main catalytic groups at the active site, which include several histidine residues that could be used to facilitate proton abstraction or metal ion binding (54–56). The insertion of ferrous iron into the sirohydrochlorin macrocycle generates siroheme (Fig. 3).

In some organisms, such as *E. coli* and *Salmonella enterica*, all three steps of siroheme synthesis, the *bis*-methylation of uroporphyrinogen III, dehydrogenation, and ferrochelation, are found within a single multifunctional enzyme called CysG (Fig. 3) (57). In essence, CysG represents a fusion between a uroporphyrinogen III methyltransferase and Met8p. The structure of CysG has been determined to show how these different domains are arranged within a dimeric structure (58). Interestingly, the enzyme was found to be phosphorylated, suggesting that phosphorylation may play a role in regulating the activity of the enzyme.

### The biosynthesis of coenzyme F_430_

**Figure 4.**
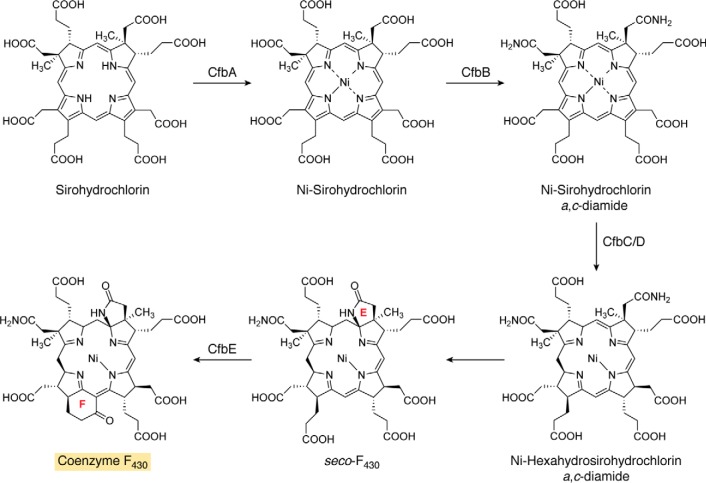
**The transformation of sirohydrochlorin into coenzyme F_430_.** The steps involved in the biosynthesis of F_430_ from sirohydrochlorin are outlined. Initially, sirohydrochlorin is chelated with nickel by the enzyme CfbA to give nickel sirohydrochlorin. Next, the two acetic acid side chains on rings A and B, the *a* and *c* side chains, are amidated in a reaction catalyzed by CfbB that also requires glutamine and ATP as substrates. This generates nickel sirohydrochlorin *a*,*c*-diamide, which acts as the substrate for the reductase system that is catalyzed by CfbC and -D. The reductase removes three double bonds from the macrocycle, which also spontaneously results in the formation of the lactam ring E, thereby generating *seco*-F_430_. The final step, mediated by CfbE, results in the formation of the cyclic hexanone ring F in another ATP-requiring process. The *shaded box* for coenzyme F_430_ coordinates with other pathway figures and the summary depiction in Fig. 14.

Coenzyme F_430_ is a nickel-containing porphinoid that plays an essential role in the reduction of methyl-coenzyme M in the production of methane gas by methanogens. Similarly, this coenzyme is also involved in anaerobic methane oxidation through a reversal of the process, which allows bacterial consortia to utilize methane as a carbon source (11). Coenzyme F_430_ is clearly based on the uroporphyrinogen III template (Fig. 1) but contains two further rings associated with the cyclization of two side chains (rings E and F). The transformation of uroporphyrinogen III into F_430_ involves methylation of rings A and B, amidation of the *a* and *c* side chains, lactam formation of the amidated *c* side chain, nickel chelation, macrocycle reduction, and cyclohexanone ring formation (Fig. 4).

Early work on the biosynthesis of coenzyme F_430_ had shown that the macrocycle was derived from precorrin-2 or sirohydrochlorin (59). However, information on the pathway remained scant, although under certain conditions a ring-F open form of F_430_, called *seco*-F_430_, could be isolated (60). Incubation of *seco*-F_430_ with a crude cell extract and ATP resulted in the formation of F_430_, suggesting that *seco*-F_430_ was the penultimate intermediate in the pathway. Progress on the biosynthesis of F_430_ biosynthesis came from analysis of the genomes of a number of different methanogens. In many methanogens, the genes encoding the enzymes for F_430_ synthesis appear to be dispersed randomly in the genome, but in a few cases the genes are functionally clustered. In several methanogens, five genes are found to localize on the genome. They encode a class II chelatase, an amidase, two reductase subunits (which also display similarity to the subunits of protochlorophyllide reductase of Chl biosynthesis), and a protein showing similarity to a ligase, MurF, involved in peptidoglycan synthesis (61, 62). The genes in the cluster were given the locus designation *cfb*, for cofactor F_430_ biosynthesis.

Recombinant production of the encoded proteins of the *cfb* cluster led to the elucidation of the F_430_ biosynthetic pathways (Fig. 4) (61, 62). The first step was shown to be the chelation of Ni^2+^ with sirohydrochlorin, mediated by CfbA. This enzyme is a small chelatase, which has a subunit molecular mass of around 12 kDa. It forms a homodimer that has a symmetrical active site. CfbA is much more active with cobalt than nickel *in vitro*, but it has a preference for nickel *in vivo*, which indicates that metal delivery and availability play important roles in ensuring that the correct metal is inserted into the correct pathway intermediates (63). The product of the reaction catalyzed by CfbA is Ni-sirohydrochlorin, which then acts as the substrate for the next enzyme in the pathway, CfbB. This enzyme amidates the *a* and *c* acetic acid side chains attached to rings A and B and requires glutamine and ATP as substrates. The enzyme is very similar to the amidase found in cobalamin biosynthesis, CobB, which amidates the same two side chains but on the corrin ring. CfbB generates Ni-sirohydrochlorin *a*,*c*-diamide, and this product is the substrate for the reductase system, which removes three double bonds from the macrocycle.

The reduction is mediated by two subunits, CfbC and CfbD. These subunits are similar not only to the reductase system that removes a double bond in Chl biosynthesis, changing the oxidation of the macrocycle from that of a porphyrin to that of a chlorin, but also to the nitrogenase subunits that are involved in nitrogen fixation. CfbC and CfbD both contain Fe-S centers and couple ATP hydrolysis with reduction of the macrocycle. Prolonged incubation of Ni-sirohydrochlorin *a*,*c*-diamide with CfbC and CfbD, together with ATP, resulted in a change in the color of the substrate, from purple to yellow, consistent with reduction of the macrocycle. An analysis of the reaction product indicated that not only had reduction taken place but also the lactam ring E had formed. However, when the reaction with the reductase subunits was incubated for shorter periods of time, then ring E was not formed (61). It may be that the formation of the lactam ring (E) is a spontaneous chemical reaction as a result of the reduction in the macrocycle. The net result, however, is the formation of *seco*-F_430_, which is the substrate for the final enzyme in the pathway, CfbE, which has similarity to the peptidoglycan ligase, MurF. In the presence of ATP, CfbE catalyzes the formation of ring F by cyclizing the propionate side chain on ring D to form a cyclohexanone structure. It is presumed that this reaction proceeds via formation of a phosphorylated intermediate to produce coenzyme F_430_.

### Biosynthesis of vitamin B_12_

Vitamin B_12_, or cyanocobalamin, is the anti-pernicious anemia factor that was first extracted from raw liver. Structurally, it is composed of a cobalt-containing ring-contracted macrocycle called a corrin (64). The cobalt ion is held not only by the four pyrrole nitrogen atoms of the macrocycle itself, but also by two further ligands that are found above (the upper or β-ligand) and below (the lower or α-ligand) the plane of the tetrapyrrole ring. In vitamin B_12_, the upper ligand is a cyano group, but this is actually a consequence of extraction when cobalamin is produced commercially (65). In biological systems, the upper ligand is normally either a methyl or an adenosyl group in methylcobalamin and adenosylcobalamin, respectively (7). The lower ligand comes from an unusual base called dimethylbenzimidazole, which is part of a nucleotide loop that is attached to the propionate side chain of ring D. Actually, cobalamin is just one member of a broader class of molecules that are referred to as either corrinoids or cobamides. The variation in corrinoid structures relates to the nature of the lower nucleotide loop and in particular the nature of the base. There are around 20 different corrinoid forms (66), of which cobalamin is just one, and it appears to be the only member that is utilized in eukaryotes. The corrinoids are unique among the vitamins in that they are made exclusively by bacteria (67). The biosynthesis of these molecules did not make the transition to the eukaryotic world, most likely because of the sheer complexity of the process, which involves about 30 steps. The catalytic properties of corrinoids appear to be associated with the ring-contracted nature of the corrin, which not only holds the cobalt tightly but also acts as an entatic state module, whereby its geometric and electronic conditions are adapted for function, in order to promote changes in the oxidation state of the metal ion (68, 69).

**Figure 5.**
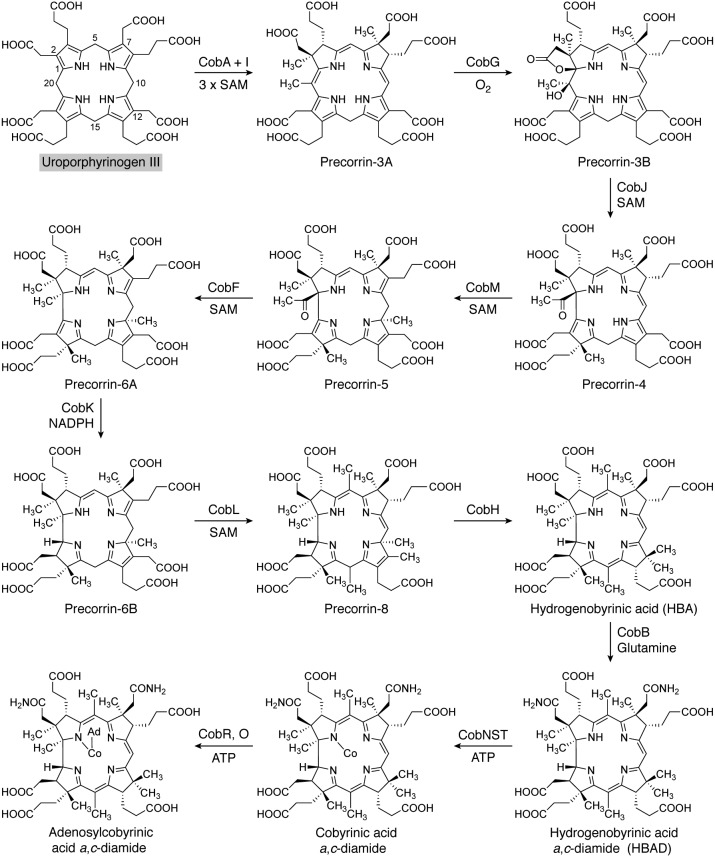
**The aerobic biosynthesis of adenosylcobyrinic acid *a*,*c*-diamide from uroporphyrinogen III.** The individual steps along the aerobic route for cobalamin synthesis are shown. Initially, uroporphyrinogen III undergoes three methylation steps at C2, C7, and C20, before hydroxylation at the C20 position generates precorrin-3B, a masked pinacol that is primed for ring contraction through rearrangement. The contraction is mediated by CobJ, which also methylates at C17. More methylations, a decarboxylation, and a mutase reaction generate the orange-colored hydrogenobyrinic acid (*HBA*) intermediate. Cobalt insertion followed by adenosylation and amidation of the side chains generates adenosylcobyrinic acid *a*,*c*-diamide, the point where the aerobic and anaerobic (see Figs. 6 and 14) pathways rejoin. The *gray shading* surrounding Uroporphyrinogen III coordinates with other pathway figures and the summary in Fig. 14.

**Figure 6.**
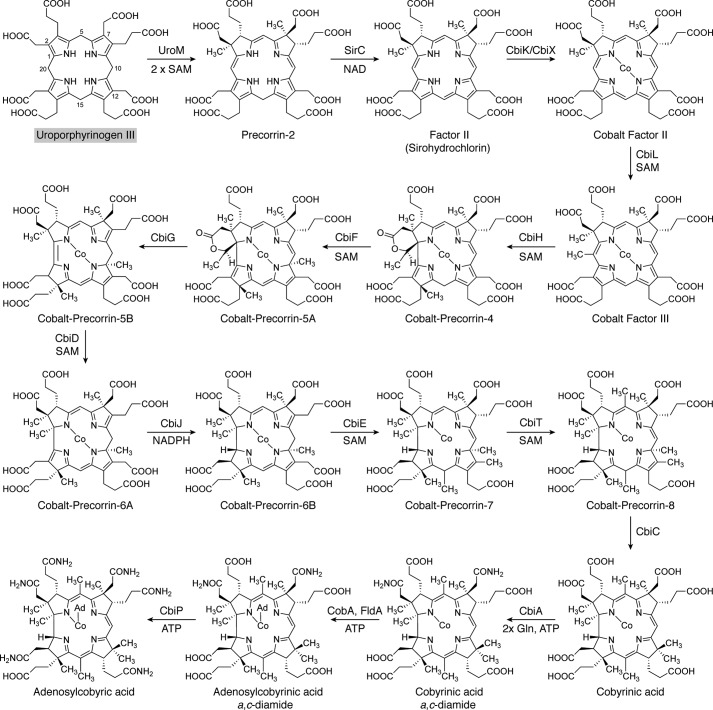
**The anaerobic biosynthesis of adenosylcobyric acid from uroporphyrinogen III.** The aerobic pathway starts with the synthesis of sirohydrochlorin, which is sometimes also referred to as Factor II. Metal insertion at this stage generates cobalt-sirohydrochlorin, which then undergoes a further methylation at C20 to give cobalt-factor III. Ring contraction is mediated by CbiH, which forms a δ-lactone in the generation of cobalt-precorrin-4. Further methylations coupled with lactone ring opening and rearrangement give rise to cobyrinic acid. Amidations together with adenosylation ultimately give rise to the formation of adenosylcobyric acid. The *shaded box* surrounding uroporphyrinogen III coordinates with other pathway figures and the summary in Fig. 14.

There are two similar although genetically distinct pathways for the biosynthesis of cobalamin (Figs. 5 and 6), which are referred to as the aerobic and anaerobic routes (70). As their names imply, the pathways differ in their requirement for dioxygen, but they also differ further in the timing of cobalt insertion (64). The two pathways diverge at precorrin-2 but rejoin at an intermediate called adenosylcobyrinic acid *a*,*c*-diamide. The final steps in cobalamin biosynthesis are similar in both pathways. The anaerobic pathway is the more common of the two routes, with the aerobic pathway being largely restricted to members of the α-proteobacteria (71).

The biosynthesis of cobalamin represents the most complex of all of the pathways for the biogenesis of modified tetrapyrroles. This reflects not only the high degree of modification that takes place on the tetrapyrrole framework but also the need to add both upper and lower ligands to the cobalt. In this respect, cobalamin is much more three-dimensional than the other members of this ring fellowship. Overall, the biogenesis of adenosylcobalamin from uroporphyrinogen III involves the addition of eight SAM-derived methyl groups, ring contraction whereby the methylated C20 carbon is extruded from the macrocycle, six amidations, a decarboxylation, cobalt insertion, aminopropanol attachment, construction and attachment of the lower nucleotide loop, and adenosylation of the cobalt ion (64). As mentioned under “The biosynthesis of siroheme,” the intermediates on the pathway are generally referred to as precorrin-*n*, where *n* refers to the number of methyl groups that have been added to the macrocycle (47). The aerobic and anaerobic pathways will be discussed separately.

### The aerobic biosynthesis of cobalamin

The aerobic pathway (Fig. 5) was elucidated in the organism *Pseudomonas denitrificans*, and genes for cobalamin biosynthesis are generally given the locus tag *cob*. Uroporphyrinogen III is methylated by CobA at positions 2 and 7 to give precorrin-2 in a reaction that requires SAM as a methyl donor (45, 72). The next enzyme in the pathway, CobI, then methylates at the C20 position to give precorrin-3A (73, 74). The following enzyme in the pathway, CobG, is a monooxygenase that requires dioxygen not only to hydroxylate the C20 position, but also to form a γ-lactone with the acetic acid side chain in ring A (75–77). This generates precorrin-3B, an intermediate containing a masked pinacol that is primed for ring contraction through a rearrangement reaction in the subsequent step. In *R. capsulatus*, the same reaction is catalyzed by a quite distinct enzyme called CobZ (78). The actual ring-contraction reaction is catalyzed by CobJ, which not only methylates the C17 position in a SAM-dependent fashion, but also contracts the macrocycle to leave the extruded methylated C20 carbon as an acetyl group attached to C1 (75, 77, 79–82). The product of this reaction is precorrin-4, which acts as the substrate for CobM that adds another SAM-derived methyl group to the macrocycle at C11, yielding precorrin-5 (83, 84). Methylation at C1 by CobF results in the loss of the extruded C20 position and produces precorrin-6A (85, 86). A reduction of the macrocycle by CobK results in the loss of a double bond in a reaction that requires NADPH as a cofactor and generates precorrin-6B (87–90).

CobL is the next enzyme in the conveyor belt and mediates methylation at C5 and C15 as well as the decarboxylation of the C12 acetic acid side chain (91–93). CobL represents a fusion between two distinct methyltransferases, with the C-terminal domain being responsible for the decarboxylation of the acetic acid side chain at C12 and the methylation at C15. The N-terminal domain performs the subsequent methylation at C5. The net result of these reactions is the synthesis of precorrin-8, which is the substrate for CobH. This enzyme is responsible for the migration of the methyl group from C11 to C12 (94, 95). In so doing, it introduces more conjugation into the macrocycle, forming an orange-colored pigment called hydrogenobyrinic acid. With all of the methylations complete, the amidation of some of the side chains then ensues. CobB amidates the *a* and *c* side chains to give hydrogenobyrinic acid *a*,*c*-diamide in a reaction that requires ATP and glutamine (96). The next step involves cobalt chelation, in which an enzyme complex formed between CobN, -S, and -T inserts cobalt in an ATP-dependent fashion to give cobyrinic acid *a*,*c*-diamide (97–99). This chelation reaction, mediated by the class I chelatase CobNST, is similar to the magnesium chelatase reaction of Chl synthesis (see below). With cobalt inserted, the upper ligand is next attached. This involves reduction of the cobalt(II) ion to a cobalt(I) species by a flavin-dependent enzyme called CobR (100, 101). The Co(I) species acts as a supernucleophile and is very unstable; it quickly reacts with the adenosyltransferase, CobO, to give adenosylcobyrinic acid *a*,*c*-diamide (102). This is the point at which the aerobic and anaerobic pathways rejoin. These steps are reviewed in more detail elsewhere (103–105).

### The anaerobic pathway

The anaerobic pathway (Fig. 6) is characterized by the way that the pathway is able to proceed in the absence of oxygen and also by the early insertion of cobalt. In contrast to the aerobic pathway, the genes for the anaerobic biosynthesis of cobalamin are given the locus tag *cbi* (67, 106). As with the aerobic pathway, the pathway initiates with the bismethylation of uroporphyrinogen III to give precorrin-2. Oxidation of the macrocycle through the removal of two protons and two electrons generates sirohydrochlorin, which is also known as factor II (57, 107, 108). This represents the substrate for cobalt insertion, whereby a class II chelatase called CbiK or CbiX inserts cobalt into the tetrapyrrole, generating cobalt-factor II (53, 55, 109, 110). CbiL methylates the C20 position of Cobalt Factor II in a SAM-dependent fashion to give cobalt-factor III (111). Ring contraction is next afforded by CbiH, which methylates at C17 and forms a δ-lactone on ring A to give cobalt-precorrin-4 (112, 113). CbiF is the next enzyme in the pathway, and, in the presence of SAM, it methylates at C11 to give cobalt-precorrin-5A (114–118). The δ-lactone ring is broken by the action of CbiG, which gives rise to cobalt-precorrin-5B and releases the extruded C20 carbon as acetaldehyde (113–115, 119). CbiD subsequently methylates at C1 to give cobalt-precorrin-6A (115, 120). Reduction of the macrocycle by CbiJ in the presence of NADH then produces cobalt-precorrin-6B (115). Decarboxylation of the acetic acid side chain attached to C12 and methylation at C15 produces cobalt-precorrin-7 in a reaction mediated by CbiT (115, 121). A further methylation at C5 by CbiE gives rise to cobyrinic acid (115). Amidation of the *a* and *c* side chains of the macrocycle by CbiA produces cobyrinic acid *a*,*c*-diamide (122). These steps are reviewed in more detail elsewhere (115, 123).

The cobyrinic acid *a*,*c*-diamide intermediate most likely acts as the substrate for the adenosylation of the cobalt ion by the adenosyltransferase. Within organisms that appear to possess an anaerobic pathway, there seem to be at least three types of adenosyltransferase (124). The first of these is called CobA, which should not be confused with the first methyltransferase of the aerobic pathway (125, 126). This enzyme is orthologous to CobO described in the aerobic pathway above in that it adenosylates the cobalt ion after it has been reduced to a Co(I) species. In the aerobic pathway, the reduction to Co(I) is apparently mediated by free flavin rather than a specific reductase. Two other adenosyltransferases, PduO and EutT, are found within pathways associated with propanediol and ethanolamine utilization, respectively, and are able to substitute for CobA (127, 128). The processes of either propanediol or ethanolamine utilization take place within specialized proteinaceous organelles called bacterial microcompartments, which both house adenosylcobalamin-dependent enzymes. Within these bacterial microcompartments, PduO and EutT are able to regenerate adenosylcobalamin when the coenzyme periodically becomes occasionally inactivated during the catalytic cycle, and hence these enzymes are not directly involved in *de novo* biosynthesis (129). In the biosynthesis of adenosylcobalamin, the adenosyltransferase results in the synthesis of adenosylcobyrinic acid *a*,*c*-diamide.

### Final stages of cobalamin biosynthesis

**Figure 7.**
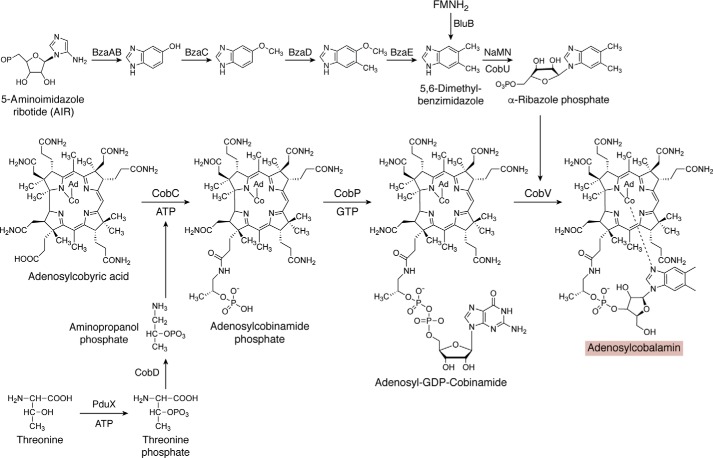
**The final stages of cobalamin biosynthesis.** Adenosylcobyric acid is converted into adenosylcobalamin through the action of three further enzymes. Initially, an aminopropanol phosphate linker is attached to the propionate side chain found on ring D to give adenosylcobinamide phosphate. Aminopropanol is itself derived from threonine. A GDP moiety is attached to the aminopropanol phosphate linker to give adenosyl-GDP-cobinamide. Finally, the GDP moiety is replaced with another nucleotide called α-ribazole, itself made from the ligation of dimethylbenzimidazole with the ribose portion of nicotinamide mononucleotide (*NaMN*). This results in the formation of adenosylcobalamin. The *shaded box* surrounding adenosylcobalamin coordinates with other pathway figures and the summary in Fig. 14.

After the synthesis of the corrin ring component by either the aerobic or anaerobic pathway, the construction of the final molecule is completed by the synthesis and attachment of the lower nucleotide loop (Fig. 7). The final amidations of the corrin macrocycle are completed by CobQ/CbiP to give adenosylcobyric acid through the addition of four amide groups from glutamine to the *b*, *d*, *e*, and *f* side chains of the tetrapyrrole framework in an ATP-dependent fashion (130, 131). This gives rise to adenosylcobyric acid. Attachment of an aminopropanol linker to the free carboxylic acid generates adenosylcobinamide in a reaction mediated by CobD/CbiB (104, 132). The aminopropanol is derived from threonine by the action of either PduX or BluE, which generates threonine phosphate (133, 134), which is decarboxylated in a pyridoxal phosphate–dependent reaction catalyzed by CobC/CobD to give aminopropanol phosphate (104, 135, 136). The adenosylcobinamide is primed for the attachment of the lower nucleotide loop by the addition of a GDP moiety, derived from GTP, to the aminopropanol in a reaction catalyzed by the homologous enzymes CobP and CobU of the aerobic and anaerobic pathways, respectively, or the nonhomologous CobY, giving rise to adenosyl-GDP cobinamide (137–139).

The unusual dimethylbenzimidazole base is made from either reduced flavin in the presence of oxygen by an enzyme called BluB (140) or from 5-aminoimidazole ribotide, a branch-point intermediate in thiamine and purine biosynthesis, in four steps (141), under anoxic conditions. The lower nucleotide is constructed by linking the dimethylbenzimidazole base to nicotinamide mononucleotide in a reaction catalyzed by CobU/T, which generates the α-ribazole phosphate (142–144). The α-ribazole phosphate then displaces the GDP moiety of the adenosyl-GDP cobinamide in a reaction catalyzed by CobV/CobS to generate adenosylcobalamin phosphate (142, 145). The phosphate is removed by a phosphatase, CobC, to give adenosylcobalamin (146, 147). More detailed reviews on the biogenesis of the lower nucleotide loop and the overall synthesis of adenosylcobalamin are found elsewhere (64, 104, 124, 148).

## Heme biosynthesis: three distinct routes from uroporphyrinogen III

**Figure 8.**
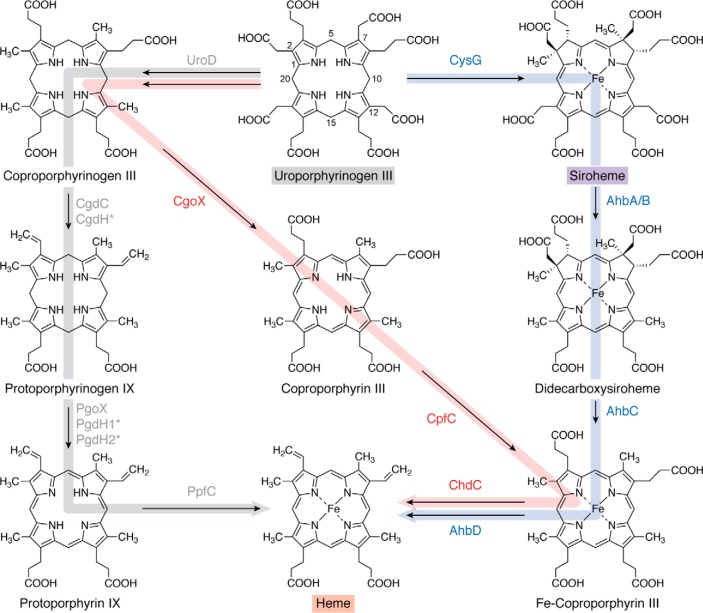
**The three routes to heme from uroporphyrinogen III.** The protoporphyrin route (*gray arrow*) involves the formation of protoporphyrin IX via coproporphyrinogen and protoporphyrinogen with the final step involving insertion of iron into protoporphyrin IX. There are aerobic and anaerobic forms of the enzymes associated with the formation of protoporphyrinogen and protoporphyrin, where the *asterisks* next to the enzyme (for CgdH, PgdH1, and PgdH2) indicate that these enzymes are found largely under anaerobic conditions. The siroheme route (*pale blue arrow*) involves the decarboxylation of siroheme to give didecarboxysiroheme, followed by the removal of the acetic acid side chains on rings A and B to give Fe-coproporphyrin before the final step, which involves the decarboxylation of the propionate side chains on rings A and B to produce heme. The coproporphyrin pathway (*dusty rose arrow*) is a hybrid between the first two routes: coproporphyrinogen is oxidized to give coproporphyrin, which is chelated with iron to give Fe-coproporphyrin. The final step is then the formation of the vinyl side chains through the decarboxylation of the propionate side chains on rings A and B. The conversion of Fe-coproporphyrin into heme is catalyzed by the same enzyme in both the siroheme and coproporphyrin pathways, although it has different names. The *shaded boxes* surrounding the names of some compounds coordinate with other pathway figures and the summary in Fig. 14.

There was a time, not that long ago, when heme biosynthesis was viewed as something quite straightforward. Now it is recognized that there are three distinct pathways (Fig. 8) for the biosynthesis of heme that are referred to as the protoporphyrin, coproporphyrin, and siroheme pathways (5).

### The ProtoIX pathway for heme (and Chl) synthesis

The ProtoIX pathway was long believed to be the only pathway for heme synthesis, involving the transformation of uroporphyrinogen III into ProtoIX in three steps prior to the insertion of iron (Fig. 8). The ProtoIX branch begins with the transformation of uroporphyrinogen III into coproporphyrinogen III through the action of uroporphyrinogen III decarboxylase (5), which decarboxylates the four carboxymethyl side chains attached to the macrocycle with the loss of four molecules of CO_2_. There is some evidence to suggest that the enzyme is able to mediate the ordered decarboxylation of the carboxymethyl side chains with ring D first followed by A, B, and C. Uroporphyrinogen decarboxylase is able to use the pyrrole rings of the macrocycle to act as an electron sink to help in the catalytic process and hence does not need any exogenous cofactor. The enzyme exists as a homodimer with a subunit molecular mass of around 40 kDa. Structural studies of the free enzyme and the enzyme with bound product have led to the idea that the mechanism may involve conserved arginine and aspartate residues acting as general acids and bases (149, 150). It has been suggested that uroporphyrinogen decarboxylase is one of the most catalytically proficient enzymes found in nature (151).

Coproporphyrinogen III next undergoes an oxidative decarboxylation of the two propionate (carboxyethyl) side chains attached to rings A and B, to generate vinyl side chains with the release of two more molecules of CO_2_. The tetrapyrrole product is protoporphyrinogen IX, and the enzyme is now referred to as coproporphyrinogen decarboxylase, CgdC (previously referred to as HemF) (5). The exact mechanism of the enzyme is unknown, and, as with the previous enzyme in the pathway, the reaction does not appear to require any exogenous cofactors or metal ions, although it does require dioxygen (152). A structure of the oxygen-dependent enzyme has been determined, and the roles of a number of amino acid residues have been investigated (153, 154). Under anoxic conditions, organisms employ an oxygen-independent version of the enzyme, which is technically a coproporphyrinogen dehydrogenase, called CgdH, but which is often referred to as HemN (155). This enzyme belongs to the radical SAM superfamily of enzymes. In this case, the enzyme utilizes SAM as a co-substrate to generate an adenosyl radical that participates in the decarboxylation process. HemN was the first radical SAM enzyme to have its structure determined (156). The reaction involves the formation of an adenosyl radical, which is able to abstract a hydrogen from the β-position of the propionate side chain, allowing elimination of the carboxylate as CO_2_ (157).

The final step in the biosynthesis of protoporphyrin IX requires the enzyme protoporphyrinogen oxidase. The enzyme catalyzes the six-electron oxidation of the macrocycle by removing six electrons and six protons with the consequent introduction of three new double bonds. The associated increase in conjugation within the system introduces color, generating the red color associated with porphyrins. As with the previous step in the pathway, there are oxygen-dependent and oxygen-independent versions of the enzyme. The best studied enzyme is oxygen-dependent and was initially named HemY but has been renamed as PgoX (5, 65). This is a flavin-dependent enzyme that exists as a homodimer with a subunit molecular mass of around 50 kDa (158). Overall, the reaction requires three molecules of dioxygen, and, because the enzyme contains a tightly but noncovalently bound FAD, it is assumed that the reaction progresses through three two-electron steps, generating three molecules of H_2_O_2_ (5). Several structures of PgoX have been determined, although the absence of either a substrate or product complex has precluded any detailed mechanistic proposal (159, 160). It is also important to note that ProtoIX is an intermediate of the Chl and BChl pathways (see below).

Two other enzymes that are also able to generate ProtoIX from protoporphyrinogen IX, but only under anoxic conditions, have been identified. Technically, these are protoporphyrinogen dehydrogenases. The first was initially called HemG but has since been renamed PgdH1 (5). This is an FMN-containing enzyme that belongs to the flavodoxin family, and it interacts with the cellular respiratory chain (161, 162). The second anaerobic enzyme was initially termed HemJ but has since been renamed PgdH2. This enzyme is the least well-characterized of the ProtoIX-forming systems, as it has not been purified. It is not known whether it is associated with any specific cofactors (163, 164). However, like PgdH1, it is thought to interact with the respiratory chain.

Heme is synthesized from ProtoIX by the insertion of ferrous iron into the porphyrin macrocycle with the loss of two protons that were previously attached to the pyrrole nitrogens. The metal-inserting enzyme is a type II chelatase and therefore does not require ATP. This enzyme is often membrane-associated, and a number of them are known to contain an Fe-S cluster, although the function of this redox center is not known (165). The best-studied ProtoIX ferrochelatase is the human enzyme, and detailed structural studies have revealed how the substrate is able to bind and induce the associated conformational changes that occur during the catalytic cycle (166).

### The siroheme pathway for heme and heme d_1_ synthesis

It has been noted by Sano and co-workers (167) that in sulfate-reducing bacteria, the two methyl groups attached to rings A and B of heme were derived from SAM, strongly indicating that heme was made from precorrin-2. Follow-up research also identified some possible intermediates on the way to heme, but the research remained incomplete. More recently, this new alternative heme pathway was finally elucidated when it was shown that the precursor for heme was actually siroheme (168). Initially, siroheme undergoes a decarboxylation of the acetic acid side chains attached on rings C and D to produce didecarboxysiroheme (Fig. 8). This reaction is mediated by a decarboxylase that is composed of two subunits, AhbA and AhbB. A structure of this enzyme has been solved, revealing how the substrate binds within the active site, in proximity to a number of highly conserved catalytic amino acid residues (169). The next step in the pathway involves a radical SAM enzyme, termed AhbC, which decarboxylates the two carboxymethyl residues on rings A and B. The mechanism underpinning this process has not been elucidated, but the reaction generates Fe-coproporphyrin, which is sometimes referred to as coproheme. The final step in the pathway involves AhbD, another radical SAM enzyme. AhbD carries out a reaction analogous to that catalyzed by CgdH, the coproporphyrinogen dehydrogenase, in that it mediates the decarboxylation of the two propionate side chains attached to rings A and B and their conversion into vinyl side chains. This siroheme pathway for heme biosynthesis is found not only in sulfate-reducing bacteria but also in members of the Archaea (168, 170). The siroheme pathway is outlined in Figs. 3 and 8.

Significantly, didecarboxysiroheme is also an intermediate for heme *d*_1_ synthesis. Heme *d*_1_ is an unusual modified tetrapyrrole that is not really a heme at all but is actually a dioxo-isobacteriochlorin (168). The genes associated with the biosynthesis of heme *d*_1_ are found within the *nir* operon in denitrifying bacteria, and include *nirD*, -*L*, -*G*, -*H*, -*F*, -*J*, and -*N*. As with heme synthesis from siroheme, heme *d*_1_ synthesis proceeds via didecarboxysiroheme, in which siroheme is decarboxylated by a combination of NirDL, -G, and -H. The propionic acid side chains attached to rings A and B of the macrocycle are extruded by the action of NirJ (171), a radical SAM enzyme that shares sequence similarity to both AhbC and AhbD. Although NirJ has been purified and has functionally been shown to be a radical SAM enzyme that catalyzes the removal of the two propionate side chains and their replacement with carbonyl groups, its reaction mechanism still has to be elucidated (172). The introduction of a double bond into the propionate side chain attached to ring D is mediated by NirN, which uses electron bifurcation to promote the dehydrogenation (173, 174).

### The coproporphyrin pathway for heme synthesis

The third variant of heme biosynthesis was identified after researchers found that some bacteria had a pathway that was not routed through either ProtoIX or siroheme (175). In essence, this pathway starts with the decarboxylation of uroporphyrinogen III by UroD to produce coproporphyrinogen as described previously. However, the next step involves the oxidation of the macrocycle to yield coproporphyrin in a reaction that is analogous to the oxidation of protoporphyrinogen to ProtoIX (*i.e.* the reaction involves the loss of six electrons and six protons) (Fig. 8). The enzyme was first thought to be a protoporphyrinogen oxidase and was initially called HemY, although now it has been renamed CpoX (5). The next step in the pathway involves ferrochelation to give Fe-coproporphyrin or coproheme. The structure of this class II ferrochelatase is very similar to that of ProtoIX ferrochelatase. Evidence suggests that this enzyme works through the distortion of the tetrapyrrole substrate to allow insertion of the metal ion (176). The final step in the pathway involves the decarboxylation of the two propionate side chains on rings A and B to produce the vinyl moieties that are found in heme. This reaction is either catalyzed in some organisms by AhbD or in others by an enzyme called HemQ, which has been renamed ChdC (5). The latter requires hydrogen peroxide as the oxidant, and crystal structures of the enzyme have led to proposed mechanisms involving key catalytic residues within the protein that are positioned adjacent to the relevant propionate side chains (177, 178). The coproporphyrin pathway is outlined in Fig. 8 and is shown to be a hybrid between the ProtoIX and siroheme routes.

## The transformation of ProtoIX into Chls: Chl *a*

**Figure 9.**
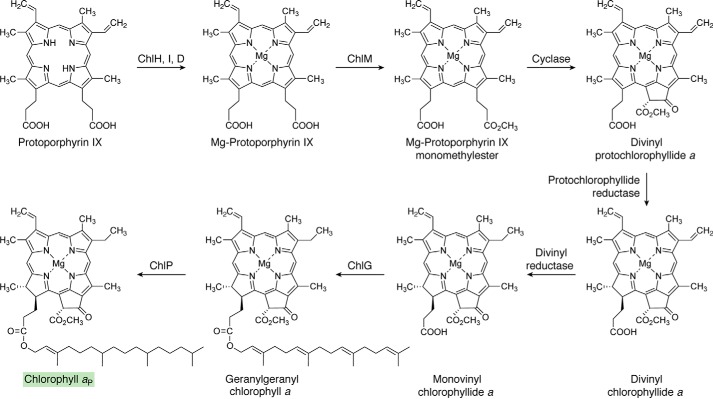
**The biosynthesis of chlorophyll *a*_P_ from protoporphyrin IX.** Magnesium insertion into ProtoIX directs the intermediate toward Chl synthesis by generating magnesium ProtoIX. This acts as the substrate for a methyltransferase (ChlM), which, together with SAM, gives rise to magnesium ProtoIX monomethyl ester. In the following reaction, the cyclase forms ring E of PChlide *a*, the C17=C18 double bond of which is then reduced, forming divinyl Chlide *a*. After reduction of one of the vinyl side chains, geranylgeraniol is attached to the propionate on ring D to form geranylgeranyl Chl *a*. Subsequent reduction of the geranylgeranyl group to phytol (*P*) gives rise to Chl *a*_P_. The *shaded box* surrounding Chl *a*_P_ coordinates with other pathway figures and the summary depiction in Fig. 14.

**Figure 10.**
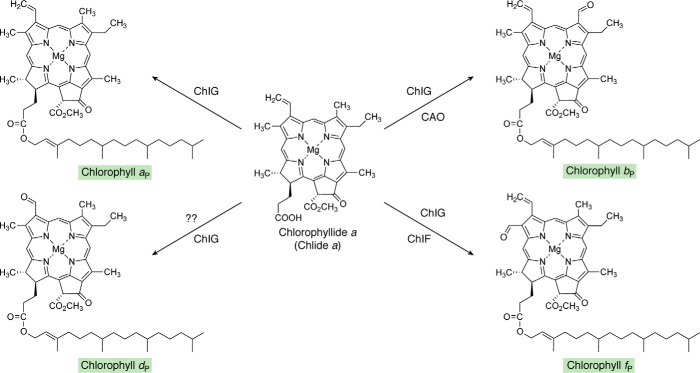
**Pathway to show the transformation of chlorophyllide *a* into other chlorophylls.** The addition of the esterifying phytol moieties to the C17 propionates is presumably catalyzed by ChlG in all cases. CAO hydroxylates the C7 methyl group twice, producing a geminal diol that spontaneously dehydrates to form the formyl group of Chl *b*. The enzyme leading to Chl *d* in *A. marina* is unknown. Chl *d* is also found in some terrestrial cyanobacteria that can photoacclimate to utilize far-red light for oxygenic photosynthesis. There is evidence suggesting that thiol compounds and/or proteins, including cysteine-rich allophycocyanins produced in far-red light, and oxygen may catalyze the formation of Chl *d*. Note that conversion of the C3 vinyl group of Chl *a* to the C3 formyl group of Chl *d* requires the loss of one carbon. The C2 formyl group of Chl *f* is introduced by a photooxidoreductase, ChlF, which is an enzyme containing Chl *a* and pheophytin *a* and which is structurally related to the D1 subunit of photosystem II. The *shaded boxes* surrounding the names of Chls coordinate with other pathway figures and the summary in Fig. 14.

Chls are the major absorbers of sunlight on Earth for photosynthesis, and consequently they supply much of the biosphere with energy. The most abundant of these pigments is Chl *a*, which is found in all oxygenic phototrophs (179), but there are also Chls *b*, *c*, and *d* and the recently discovered Chl *f* (Figs. 9 and 10) (180). Chls form a structurally and functionally distinct group within the porphyrin family, characterized by the presence of a fifth ring, the isocyclic “E” ring, and an alcohol, usually phytol, esterified at the C17^3^ position. A central magnesium ion is bound via coordinating bonds to the four central nitrogen atoms of the tetrapyrrole. The pyrrole rings of Chls form an extended system of conjugated bonds that confers strong absorption, not only in the blue-violet region of the electromagnetic spectrum, a property shared with many porphyrins, but crucially also in the red. Chls possess other structural features that amplify absorption in the red and also extend it toward 750 nm; these include the carbonyl on ring E, and various side chains at C1–4; the most red-shifted pigment, Chl *f*, has C2 formyl and C3 vinyl substituents (180). Chls are more than just light absorbers, and when they are situated in an appropriate protein environment, they acquire another function, redox activity, which is crucial for their central role in reaction center complexes (179).

The biosynthesis of Chls is initiated when magnesium (Mg^2+^), rather than the Fe^2+^ used for heme biosynthesis, is inserted into the ProtoIX macrocycle. The large, multisubunit Mg chelatase complex catalyzes this thermodynamically challenging reaction, in which the free energy of hydrolyzing ∼15 ATP molecules (181) is used to drive conformational alterations in the complex, likely associated with distortion of the ProtoIX and selective depletion of the hydration shell surrounding the Mg^2+^. The ∼140-kDa ChlH subunit binds the porphyrin substrate (182), and the ∼40-kDa ChlI and ∼80-kDa ChlD subunits belong to the ATPases associated with various cellular activities (AAA^+^) superfamily. The current view is that ChlD is the physical and mechanical link between the ChlI AAA+ motor and ChlH, which houses the site of metal ion chelation (183).

Although the ChlHID subunits form the catalytic core of magnesium chelatase and are sufficient for *in vitro* activity, plants and cyanobacteria also require a small (∼26-kDa) soluble protein, Gun4, and make little Chl in its absence (184). Gun4 lowers the magnesium concentration required for chelatase activity at low porphyrin concentrations (185) and substantially enhances the catalytic rate, by at least 10-fold (181). There are structures of apo-ChlH (186), apo-Gun4 (185, 187, 188), and *Synechocystis* sp. PCC 6803 Gun4 bound to both deuteroporphyrin and Mg-deuteroporphyrin (189); however, structures of subcomplexes, and the entire Mg chelatase complex at various stages of the catalytic cycle, will be required for a complete characterization of this important enzyme complex.

The next step in Chl biosynthesis involves esterification of the propionate side chain attached to ring C with a methyl group, forming Mg-ProtoIX monomethylester (Fig. 9) (190). This reaction, which requires SAM as the methyl donor, is catalyzed by the enzyme *S*-adenosyl-l-methionine Mg-ProtoIX methyltransferase (ChlM) (191). Steady-state and transient kinetic analyses of ChlM, produced heterologously in *E. coli*, show that the reaction proceeds by a random-binding mechanism that forms a ternary ChlM-SAM-Mg-ProtoIX complex (192). Intriguingly, the magnesium chelatase subunit ChlH accelerates the formation and breakdown of an intermediate in the catalytic cycle of ChlM (193). Another link between the two first committed steps of Chl biosynthesis is suggested by Gun4, which could play a role in trafficking Mg-ProtoIX from ChlH to ChlM; the propionate group of Mg-deuteroporphyrin that is methylated by ChlM has been observed to protrude from the binding cleft of Gun4, potentially exposing it to ChlM (194).

The methylated propionate side chain on ring C is used to form the isocyclic fifth (E) ring (Fig. 9), in a series of reactions catalyzed by Mg-ProtoIX monomethylester (oxidative) ring cyclase (cyclase) that produce 3,8-divinylprotochlorophyllide (divinyl-PChlide). The formation of ring E is accompanied by a transition to a green color, and this structural change creates an absorption band at 630 nm, a crucial step in the eventual formation of Chl *a* with strong absorption at 665 nm in methanol. Experiments with ^18^O-labeled molecular oxygen showed its direct incorporation into the carbonyl group of the isocyclic ring (195), so this enzyme is referred to as an oxidative, aerobic, or O_2_-dependent cyclase. There is also an O_2_-independent cyclase, BchE, which incorporates oxygen donated from water (196). Although BchE homologs (termed ChlE) have been found in some cyanobacteria (197), the O_2_-independent cyclase is found mainly in anoxygenic phototrophic bacteria, and it will be described under “The biosynthesis of BChls *a*, *b*, and *g*.” Pinta *et al.* (198) assigned AcsF (aerobic cyclization system Fe-containing subunit) in the purple betaproteobacterium *Rubrivivax gelatinosus* to the O_2_-dependent reaction, and homologs were subsequently found in all oxygenic photosynthetic organisms investigated. These include the genes *Crd1* and *Cth1* in *Chlamydomonas reinhardtii* (199, 200), *sll1214* and *sll1874* in *Synechocystis* sp. PCC 6803 (201, 202), *Chl27* in *Arabidopsis* (203), and *Xantha-I* in barley (204). Three classes of O_2_-dependent cyclases have been identified: in betaproteobacteria, AcsF is sufficient, whereas oxygenic phototrophs require an auxiliary subunit, Ycf54 (205), and alphaproteobacteria also require BciE (206). Kinetic and structural characterization of the O_2_-dependent cyclase await the availability of sufficient quantities of pure, active protein.

Following formation of the E ring by the cyclase, reduction of the C17=C18 double bond of ring D further alters the π-electron system and produces chlorophyllide (Chlide), with a stronger, more red-shifted absorption transition approaching that of the final pathway product, Chl *a* (Fig. 9). Nature has discovered two completely different ways to achieve this reduction: in one, a light-dependent reaction is catalyzed by NADPH:protochlorophyllide oxidoreductase (LPOR) (207, 208); in the other, reduction of the C17=C18 double bond is catalyzed by a dark-operative protochlorophyllide reductase (DPOR), consisting of ChlL, ChlN, and ChlB subunits that display similarity to the components of nitrogenase (209). The ability to trigger the catalytic cycle with short pulses of light has led to a number of kinetic studies (210–212), and recently the structure of LPOR has been reported (208). The phylogenetic distribution of LPOR and DPOR is interesting: anoxygenic photosynthetic bacteria contain only DPOR; cyanobacteria, green algae, mosses, and most gymnosperms possess both LPOR and DPOR; and angiosperms (flowering plants) contain only LPOR. The half-life of DPOR rapidly declines upon exposure to oxygen (213), and possibly this enzyme could not tolerate the advent of oxygenic photosynthesis (214, 215). Instead, bacteria capable of oxygenic photosynthesis are thought to have adapted to the increasing oxygen content of the atmosphere by acquiring the oxygen-insensitive, light-dependent LPOR (216). A similar consideration may apply to the adoption of the oxygen-dependent MgPME cyclase (217).

The majority of Chls have a single vinyl group at the C3 position and an ethyl group at C8. The 3,8-divinyl Chlide formed by protochlorophyllide reductase is reduced by 8-vinyl reductase, also known as divinyl reductase, forming 8-ethyl Chlide (monovinyl-Chlide) (Fig. 9). Two groups independently isolated the first gene encoding divinyl reductase, AT5G18660, by characterizing *Arabidopsis thaliana* mutants that accumulate divinyl-Chl (218, 219). Cell extracts from *E. coli* overexpressing the AT5G18660 gene catalyzed the conversion of divinyl-Chlide to monovinyl-Chlide (218). Homologs of the AT5G18660 gene are found in higher plants, green algae, some green sulfur bacteria (GSB), and some purple bacteria, and *Synechococcus* spp., but not in red algae, filamentous anoxygenic phototrophs, or freshwater cyanobacteria (220–222). The homologous gene encoding divinyl reductase in phototrophic bacteria was renamed as *bciA* (220), and homologs have been confirmed by genetic mutation (*Chlorobaculum tepidum*, rice, and *Rhodobacter sphaeroides*), genetic complementation (*R. sphaeroides*) and recombinant divinyl reductase assays (*C. tepidum* and rice) (220, 224, 225). A second type of divinyl reductase, termed BciB, is found in many cyanobacteria, higher plants, green algae, some GSB, some purple bacteria, and some filamentous anoxygenic phototrophs (226–228). Although plant and green algal genomes contain homologs of both *bciA* and *bciB*, most cyanobacterial genomes only contain homologs of one gene, either *bciA* or *bciB*. An exception is found in the marine cyanobacterium *Acaryochloris marina*, which has both forms of divinyl reductase (229). Whereas BciA uses NADPH (220) as the reductant, BciB from GSB contains an FAD cofactor and [4Fe4S] clusters and uses ferredoxin as the reductant for this reaction (228).

The final steps of Chl *a* biosynthesis involve attachment of a C20 isoprenoid alcohol, geranylgeraniol, to the C17 propionate side chain of monovinyl-Chlide *a* and then its reduction to phytol. In the reaction sequence shown in Fig. 9, geranylgeraniol diphosphate is attached to monovinyl-Chlide *a*, and then it is subsequently reduced to phytol. However, it is also possible that phytol diphosphate is attached to monovinyl-Chlide *a* following prior reduction of the free alcohol (230). The enzyme that catalyzes this esterification of the C17 propionate is Chl synthase, ChlG, which is predicted to be an intrinsic membrane protein of ∼42 kDa (231). ChlG catalysis proceeds via a ping-pong mechanism in which geranylgeraniol diphosphate (or phytol diphosphate) binds first to the enzyme and causes a conformational change in ChlG, allowing it to bind Chlide, the second substrate (232). Residues 88–377 are catalytically active, and Arg-91, Arg-161, and Cys-109 are critical for the synthase activity (233). The other enzyme involved is geranylgeranyl reductase, ChlP, which in *A. thaliana* is capable of catalyzing the stepwise reduction of free geranylgeranyl diphosphate into phytol diphosphate as well as the reduction of monovinyl-Chlide *a*_GG_ into monovinyl-Chlide *a*_P_, namely Chl *a*_P_ (234, 235). Although phytylation exerts only a small influence on the spectroscopic properties of the monovinyl-Chlide *a* substrate (236), there is a significant increase in hydrophobicity, which is crucial for the assembly and function of Chls within membrane-intrinsic, light-harvesting, and reaction center complexes. The completion of the Chl biosynthetic pathway necessitates the handover of this pigment from the membrane-intrinsic Chl synthase to the machinery for synthesis of nascent proteins and their insertion into the membrane bilayer. Co-purification of ChlG with the YidC insertase (237) indicates a link between Chl biosynthesis and membrane assembly, and the additional presence of Ycf39, HliD, and HliC proteins suggests that an element of photoprotection is incorporated into the synthase complex.

The Chl biosynthetic pathway is usually studied one step at a time, for ease of interpreting kinetic and structural data, but within the cell, these enzymes must function while bathed in light and in the presence of oxygen generated by photosystem II. It is possible that a large multienzyme assembly could channel photolabile biosynthetic intermediates between active sites, minimizing their exposure to light and oxygen. The heterologous assembly of the Chl biosynthesis pathway in *E. coli* provides a platform for investigating the physical and mechanistic coupling between pathway enzymes (238). This achievement also demonstrates that, after decades of research, all reactions necessary for the biosynthesis of Chl *a* have now been identified.

## Extension of the pathway beyond Chlide *a*/Chl *a*

Following the evolutionary invention of Chl *a*, high concentrations of which absorb light completely in the 400–700-nm spectral range, light probably became a limiting resource in many niches. To deal with the problem of harvesting light for photosynthesis in competition with Chl *a*, bacteria, plants, and algae evolved other pigments (*e.g.* carotenoids and bilins) as well as the ability to produce Chls and BChls with different absorption properties. Fifteen major Chl/BChl species, with different tetrapyrrole headgroups, are known, and additional molecular diversity occurs because of esterification by different alcohols and/or the occurrence of compounds with Mg^2+^, Zn^2+^, or no chelating metal ion (pheophytins). As can be seen from Fig. 10, Chlide *a* represents a hub intermediate (*i.e.* a central intermediate) for biosynthesis of Chls *a*, *b*, *d*, and *f* (220, 222, 239). More generally, nearly all Chls and BChls are derived from one of the two central intermediates, divinyl-Chlide *a* and Chlide *a*. Note that the order of the terminal reactions in cells is probably not rigid, because divinyl reductases have somewhat relaxed substrate specificity and can act before or after D-ring reduction by PChlide reductase.

**Figure 11.**
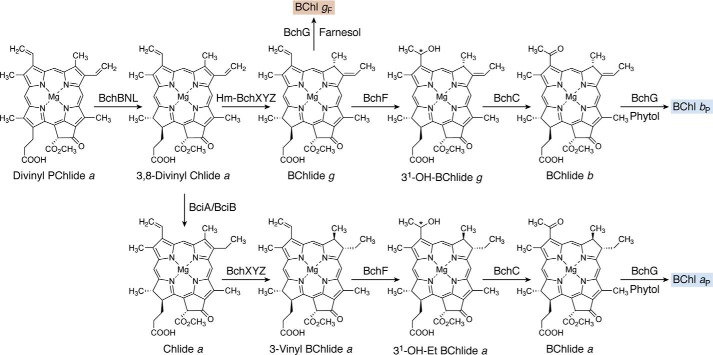
**Synthesis of bacteriochlorophylls *a*, *g*, and *b* from divinyl-protochlorophyllide *a*.** There are two types of Chlide *a* oxidoreductases. The type found in *R. sphaeroides* and most other anoxygenic phototrophs converts Chlide *a* into 3-vinyl BChlide *a*. However, organisms such as *Heliobacterium modesticaldum* (*Hm*) that produce BChl *g*_F_ or *Blastochloris viridis* that produce BChl *b* have an enzyme that converts 3,8-divinyl Chlide into BChlide *g*, which has an ethylidene side chain at the C8 position. BChl *a* and *b* are usually esterified with phytol by the BChl synthases (BchG) that occur in those organisms. However, BChl *g* is esterified with farnesol by the Bchl *g* synthases that occur in heliobacteria. Note that divinyl PChlide is also the precursor for the synthesis of the family of pigments known as Chl *c*. For additional details, see section “Extension of the pathway beyond Chlide *a*/Chl *a*.” The *shaded boxes* surrounding the names of some compounds coordinate with other pathway figures and the summary in Fig. 14.

In about half of the cases, the pathway beyond Chlide *a*/Chl *a* was extended to produce compounds with enhanced absorption in the blue region of the solar spectrum. For example, Chl *b*, which is produced by adding a single enzyme beyond Chlide *a* (Fig. 10), is an example of an extension of the main pathway to enhance absorption of blue light. On the other hand, the loss of a single enzyme can also be sufficient to account for a unique Chl product with enhanced blue light absorption. The absence of 8-vinyl reductase activity (BciA or BciB) leads to strains producing divinyl-Chl *a* (or divinyl-Chl *b*) (Fig. 11). This minor chemical difference bathochromically shifts the Soret absorbance band slightly compared with that of Chl *a*, which allows cells producing these divinyl-Chls to absorb slightly different wavelengths of blue-green light than Chl *a* or Chl *b*. The exception to the hub compounds described above is the small family of Chls known collectively as Chls *c*. Chl *c* derivatives are apparently produced as derivatives of PChlide (240) and have very strong absorption in the blue but absorb much more weakly in the red (240, 241). Unlike most other Chls, members of the Chl *c* family do not have an esterifying alcohol on the carboxyl group of the C17 propionate, and this side chain is oxidized to contain a double bond in some of the family members (240). BChl *e* and *f* of GSB are also specialist molecules for absorbing blue light, but they only gain this emergent property after the formation of supramolecular aggregates in chlorosomes. To date, BChl *f* is not known to occur naturally, but mutants that can produce BChl *f* have been produced and studied in the laboratory (242).

Solar radiation reaching Earth contains only ∼10% fewer photons between 700 and 1100 nm than visible light (400–700 nm), so it is not surprising that many organisms evolved Chls/BChls to use those wavelengths for photosynthesis as well. Two Chls (Chl *d* and Chl *f*) and five BChls (BChl *a*, *b*, *c*, *d*, and *g*) have enhanced absorption principally in the far-red/near-IR wavelength regions. All of these compounds are made by pathway extensions leading from divinyl-Chlide *a* or Chlide *a.* In some cases, a single enzyme can again account for the production of a new Chl species derived from Chlide *a* with modified light-harvesting potential. In other cases, multiple steps were required to produce a compound with beneficial and new light-harvesting properties.

## The biosynthesis of Chls *b*, *d*, and *f*

The addition of a formyl group at the C7 position of Chl *a*, forming Chl *b* (Fig. 10), distorts the π-electron system of the macrocycle and draws electron density along the Q*_x_* axis at the expense of the Q*_y_* transition. The Q*_y_* axis runs from ring A to ring C, and the Q*_x_* axis runs from ring B to D; see Fig. 1 for the labeled rings in the analogous uroporphyrinogen III molecule. This modification attenuates and blue-shifts the Q*_y_* absorption band while red-shifting the Soret band (243), which complements the absorption of Chl *a*. Thus, the major light-harvesting complex of plants, LHCII, which binds nearly equal proportions of Chl *a* and *b* (244), acquires an enhanced capacity for absorbing light. Chl *a* oxygenase (CAO) was discovered by studying mutants of *C. reinhardtii* unable to synthesize Chl *b* (245). An *in vitro* assay for CAO was devised with Chlide *a* as a substrate, which showed that recombinant CAO from *A. thaliana* catalyzes conversion of the C7 methyl to a formyl (246), a reaction that requires dioxygen (247). The reaction appears to proceed by producing 7^1^-OH-Chlide *a* as an intermediate (248). Collectively, these observations strongly suggest that CAO catalyzes consecutive hydroxylations of the C7 methyl group to produce a *geminal* diol intermediate, which can spontaneously dehydrate to produce Chlide *b*. Although a detailed structural and mechanistic model is still lacking for CAO, the gene sequence encodes putative binding domains for a Rieske-type [2Fe-2S] center and for a mononuclear nonheme iron. The true substrate could be Chl *a*, rather than Chlide *a* (249), which raises the intriguing possibility that CAO could use both free and protein-bound Chl *a* as substrates.

Chl *d* was first described as a minor pigment associated with red algae in 1943 (250). Structurally, it is similar to Chl *a*, except the 3-vinyl group is replaced by a formyl group (Fig. 10), which causes this pigment to absorb maximally in methanol at 697 nm, a red shift of about 35 nm (243, 251). Although initially suspected to be an artifact, the discovery of *Acaryochloris marina* in 1996, which has Chl *d* as its major Chl with only minor amounts of Chl *a*, validated its occurrence in cyanobacteria (252, 253). Subsequent studies showed that the original report was probably due to epiphytic cyanobacteria that grew on the underside of the red algal fronds (254, 255). More recently, small amounts of Chl *d* have been found in diverse terrestrial cyanobacteria that also synthesize Chl *f* when grown in far-red light (FRL) (256–260). In these organisms, Chl *d* is specifically associated with FRL-photosystem II but is not present in the FRL-photosystem I complexes (259, 260). It has been suggested that Chl *d* may be a component of the electron transport chain in such organisms (260).

Numerous studies have been conducted to identify the enzyme(s) responsible for the synthesis of Chl *d* in *A. marina*, but to date, the enzyme(s) remains unknown. Labeling studies have suggested that the oxygen atom in the 3-formyl group is derived from dioxygen and that the precursor is Chlide *a*/Chl *a*, but otherwise little is known (261). *In vitro* studies indicate that Chl *d* can easily be produced when Chl *a* is incubated with thiol reagents (*e.g.* thiophenol or 2-mercaptoethanol) in the presence of oxygen (262), and one report found that the Chl *a* could be converted into Chl *d* in the presence of the thiol protease, papain (263). A reaction mechanism involving thiyl radicals was suggested to play a role in Chl *d* synthesis catalyzed by thiophenol (262). Very recently, mutants unable to synthesize the FRL-specific allophycocyanins associated with growth in FRL were found to be strongly depleted or even devoid of Chl *d* (264). Unlike most other phycobiliproteins, these allophycocyanin subunits contain 2–4 cysteine residues that are not involved in covalent binding of phycocyanobilin. It is possible that these thiol-rich and abundant proteins play a role in producing the small amount of Chl *d* required for growth in FRL, just as papain does *in vitro* (264). Such a mechanism would likely not function in *A. marina*, which produces minimal amounts of phycobiliproteins that do not contain such extra cysteine residues; however, some other sulfhydryl-containing small molecule or cysteine-containing protein could play a role in Chl *d* synthesis in this cyanobacterium.

Chl *f* was discovered less than 10 years ago in a cyanobacterium derived from a stromatolite found in Sharks Bay, Australia (180, 243, 265). This Chl absorbs maximally at about 707 nm in organic solvents, the longest value for any naturally occurring chlorin described to date (251, 266). Chl *f* only differs from Chl *a* by having a formyl group at C2 rather than a methyl group (Fig. 10). The oxygen atom of the formyl group has been reported to be derived from dioxygen, but it is unclear whether Chlide *a* or Chl *a* is the precursor (267). Chl *f* synthase was discovered to be one of the 20 genes in the far-red light photoacclimation gene cluster by deletion mutagenesis in two cyanobacteria (259). Surprisingly, Chl *f* synthase is related to the PsbA (D1) core subunit of photosystem II and is a member of the so-called super-rogue PsbA subfamily. These PsbA paralogs are highly divergent members of the PsbA superfamily and lack the essential amino acid residues associated with ligation of the Mn_4_Ca_1_O_5_ water oxidation complex. Deletion of the *chlF* (formerly *psbA4*) gene produced a mutant that was unable to synthesize Chl *f* and that was unable to grow in FRL (259). Heterologous expression of *chlF* genes in a cyanobacterium that normally synthesizes only Chl *a* showed that this strain gained the ability to synthesize Chl *f*, even if photosystem II was inactivated by deletion of the two copies of the *psbD* genes in the organism (268). PsbA binds Chl *a*, β-carotene, and pheophytin *a*, and, likewise, purified Chl *f* synthase is a homodimer that binds these same cofactors. Furthermore, the Chl *f* biosynthetic reaction is light-dependent and sensitive to 3-(3,4-dichlorophenyl)-1,1-dimethylurea, which suggests that it may act by transferring electrons to plastoquinone (259, 268). Although it was initially hypothesized that the substrate Chl *a* or Chlide *a* molecule might bind in a pocket formed by the missing Mn_4_Ca_1_O_5_ cluster, more recent site-specific mutagenesis experiments suggest that the substrate may bind in a peripheral site analogous to the Chlz_D1_ binding site of photosystem II.^5^ To date, no *in vitro* enzymatic assay system has yet been developed for this enzyme. The development of such a system will be the key to learning more about this interesting reaction.

## The biosynthesis of BChls *a*, *b*, and *g*

Some of the biosynthetic steps leading to BChls *a*, *b*, and *g* are similar to those for the Chl *a* pathway but use enzymes that have evolved to function in the oxygen-limited, even anoxic, conditions experienced by purple phototrophic bacteria. Thus, the BChl pathway (Fig. 11) includes an O_2_-independent E-ring cyclase and a DPOR reminiscent of nitrogenase, which produces Chlide *a*, and a paralogous enzyme, Chlide *a* oxidoreductase (COR), that reduces it to form bacteriochlorophyllide (BChlide) *a* (222). As noted above, divinyl-Chlide *a* can be considered as a hub for biosynthesis of nearly all Chls and BChls, and indeed by switching the enzymes that feed off this centrally important pigment, it is possible to convert a phototrophic bacterium, *R. sphaeroides*, to the synthesis of Chl *a* (269).

The first committed step of BChl biosynthesis is catalyzed by magnesium chelatase, but there are some aspects of this enzyme complex that differ from the Chl-specific version, notably the absence of an analog of Gun4, which is so important for chelation *in vivo* (184), and the presence of an Fe/S cluster in the BchH subunit (270). Although the role of this cluster is not known, it probably has some regulatory significance related to the need for bacteria such as *R. sphaeroides* to control levels of BChl in response to oxygen concentration. It is interesting that the ferrochelatase in this bacterium, which catalyzes the formation of heme at the other step at this biosynthetic branch point, may also have an FeS cluster.^6^ The ferrochelatase from oxygenic phototrophs is no less important, given the need to control partitioning between the heme/bilin and Chl branches of tetrapyrrole biosynthesis, but in this case, the catalytic core is attached to a hydrophobic C-terminal, CAB (Chl *a*/*b*-binding) domain with a putative Chl-binding motif (271, 272). Clearly, this partitioning is too important to be left solely to the respective activities and catalytic mechanisms of the magnesium and Fe chelatases, and in *R. sphaeroides*, the PufQ protein binds to ferrochelatase and makes sufficient ProtoIX available for BChl synthesis (273).

The oxygen-independent E-ring cyclase, found in all bacteria that synthesize BChls, is encoded by *bchE* (274), and it obtains the required oxygen for the keto group of the E-ring from water (196). Cobalamin is required for the oxygen-independent cyclase (167), and in the mechanism proposed for the anoxic reaction, adenosylcobalamin forms an adenosyl radical, which in turn leads to the formation of the 13^1^-radical of MgPME. Withdrawal of an electron forms a 13^1^-cation, which reacts with water to form the 13^1^-hydroxy intermediate of MgPME. Finally, the withdrawal of three hydrogen atoms leads to the eventual cyclization and formation of PChlide. BchE, which is a member of the radical SAM superfamily, contains an Fe/S cluster that may be involved in the initial electron transfer steps of the mechanism (275).

All photosynthetic organisms except angiosperms have the DPOR, which catalyzes reduction of the C17=C18 double bond using a heterotrimeric complex resembling nitrogenase consisting of BchL, BchN, and BchB subunits (Fig. 11). Ferredoxin functions as an electron donor, and the enzyme consists of a BchL homodimer as a reductase component that contains an oxygen-sensitive [4Fe-4S] cluster and a BchN-BchB heterotetramer as the catalytic component. The crystal structure of BchL from *R. capsulatus*, determined with bound MgADP to 1.6 Å resolution (276), shares overall structural similarity with the Fe protein of nitrogenase, including the [4Fe-4S] cluster and the nucleotide-binding sites. Crystal structures of the BchN-BchB protein from *R. capsulatus* were solved both in the PChlide-bound and PChlide-free forms at 2.3 and 2.8 Å resolution, respectively (277). Subsequently, the X-ray crystallographic structure of the 360-kDa BchL_2_-(BchN-BchB)_2_ transition state complex was solved with bound PChlide (278). The proposed reaction mechanism of DPOR involves ATP-dependent electron transfer from the [4Fe-4S] cluster of BchL to the [4Fe-4S] cluster of (BchN-BchB)_2_, which houses the active site, where coupled electron and proton transfers reduce the C17=C18 double bond of PChlide.

BChl *a* has an ethyl group at C8, and the variety of 8-vinyl reductases reflects the importance of ensuring the proper formation of this side chain. Some early reports suggested that the vinyl reductase of *R. capsulatus* was encoded by *bchJ* (279), but inactivation of *bchJ* in *C. tepidum* does not block the reduction of the C8 vinyl group but did result in the excretion of large amounts of divinyl-PChlide into the growth medium (220). This phenotype suggests that BchJ might play a role as a chaperone in the BChl biosynthetic pathway, but this has not been verified experimentally. *R. sphaeroides* has BciA (224), and a second type of 8-vinyl reductase, BciB, is found in GSB (227, 228), some purple bacteria, and some filamentous anoxygenic phototrophs (221, 226). C8-vinyl Chlide, rather than C8-vinyl PChlide, is the preferred substrate for both BciA and BciB (280, 281). BciB from the GSB *Chloroherpeton thalassium* was produced recombinantly in *E. coli*; it has two [4Fe-4S] clusters and an FAD cofactor, and *in vitro* assays showed that BciB is a ferredoxin-dependent 8-vinyl reductase (228). Intriguingly, COR (Chlide *a* oxidoreductase), which reduces the C7=C8 double bond of Chlide *a*, also possesses 8-vinyl reductase activity (281), so the COR-type 8-vinyl reductase represents a third class of this enzyme.

The hallmark of BChls is their absorption in the near IR, which in organic solvents is bathochromically shifted by about 100 nm with respect to Chl *a*. Two major modifications of the 8-ethyl Chlide *a* macrocyle are responsible for this absorption shift. The 3-vinyl moiety of ring A is converted to a 3-acetyl group, and the C7=C8 double bond of ring B is reduced. Together, these changes alter the distribution of the π-electron system, away from the B ring and toward the ring A–C (Q*_y_*) axis, promoting strong absorption that is bathochromically shifted from 661 to 734 nm for BChlide *a*. The enzymes involved in these steps are as follows: COR (BchXYZ) catalyzes the reduction of the C7=C8 double bond of ring B; 3-vinyl BChlide *a* hydratase (BchF) is responsible for the hydroxylation of the 3-vinyl group; and 3-hydroxyethyl (3HE)-BChlide *a* dehydrogenase (BchC) converts the 3HE group to the 3-acetyl group (Fig. 11) (222). The order of the two steps catalyzed by COR and 3-vinyl BChlide *a* hydratase was shown to be interchangeable by the isolation of a BChl intermediate, 3-vinyl BChlide *a* (282).

COR, as a second nitrogenase-like enzyme in (B)Chl biosynthesis, shares many similarities with DPOR, not only in the reaction chemistry but also in enzyme composition. This similarity extends to the CfbC/D proteins that catalyze reduction of Ni^2+^-sirohydrochlorin *a*,*c*-diamide to Ni^2+^-hexahydrosirohydrochlorin *a*,*c*-diamide (283), a step in the biosynthesis of coenzyme F_430_ (see above). Genetic studies of *R. sphaeroides* and *R. capsulatus* revealed the three genes, *bchX*, *bchY*, and *bchZ*, that encode COR (274, 284, 285). *R. capsulatus* COR was reconstituted with purified BchX and BchY-BchZ proteins using the same biochemical approaches applied to reconstitute DPOR, and, as expected, COR requires ATP and dithionite for activity (286). Furthermore, the recombinant COR from *Roseobacter denitrificans* was used to study the substrate binding and catalytic mechanism (287). COR is able to use substrates with modifications on rings A, C, and E, but not with modifications of the C17-propionate group. EPR revealed the presence of a [4Fe-4S] cluster in (BchX)_2_ and a second [4Fe-4S] cluster in the (BchY/BchZ)_2_.

Formation of the 3-acetyl group of BChl *a* proceeds through the action of a hydratase and then an oxidation/dehydrogenase step (Fig. 11); the participation of a hydratase was shown by ^18^O-labeling and MS (196). 3-Vinyl BChlide *a* hydratase is encoded by the *bchF* gene (284, 288). Biochemical characterization of this enzyme, which was produced by heterologous overexpression of *C. tepidum bchF*, revealed an integral transmembrane protein that converts Chlide *a* to 3HE Chlide *a* (289). BchF produces primarily *R*-stereochemistry at the chiral C3^1^ position. This is essential because the next step in the pathway is catalyzed by an NAD^+^-dependent dehydrogenase; such enzymes require specific substrate chirality for their action. The oxidation of the 3HE to form a 3-acetyl group is catalyzed by the product of the *bchC* gene (290, 291). The *C. tepidum bchC* was overexpressed in *E. coli* and subsequently purified as a soluble BchC-NAD^+^ complex, which was shown to have broad substrate specificity: modification of the E ring is tolerated, the central Mg^2+^ can be omitted or replaced with Zn^2+^, and a nonreduced B ring is also accepted (289).

The final steps of BChl biosynthesis are similar to those for the Chl pathway, namely the reduction of geranylgeraniol and its attachment to the C17 propionate side chain of BChlide *a* (Fig. 9). Geranylgeranyl-reductase (BchP) catalyzes the NADPH-dependent stepwise reduction of free geranylgeranyl diphosphate into phytol diphosphate as well as the reduction of BChlide *a*-geranylgeranyl into BChlide *a*-phytyl, namely BChl *a* (292, 293). The attachment of either geranylgeranyl diphosphate or phytol diphosphate to BChlide is catalyzed by BChl synthase (Fig. 11). Early genetic studies showed that *bchG* encodes BChl synthase in *R. sphaeroides* and *R. capsulatus*, as mutations of this gene accumulate BChlide *a* and lack BChl *a* (294, 295). Subsequently, BChl synthase assays were developed using extracts from *E. coli* overexpressing *bchG* (296, 297). (B)Chl synthases exhibit a high degree of specificity for the tetrapyrrole substrate; ChlG from Chl-containing organisms can only utilize Chlide *a* and not BChlide *a*, whereas BchG from BChl-producing organisms can only utilize BChlide *a* and not Chlide *a* (297–299). Competitive inhibition of the ChlG of *Synechocystis* sp. PCC 6803 by BChlide *a* or of BchG of *R. sphaeroides* by Chlide *a* was observed, suggesting that the active sites of ChlG and BchG are structurally similar (300). Ile-44 of *Synechocytis* sp. PCC 6803 ChlG and Phe-28 of *R. sphaeroides* BchG were found to be responsible for the tetrapyrrole substrate specificity based on the experimental evidence that the ChlG I44F mutant has activity with BChlide *a*, whereas the BchG F28I mutant has activity with Chlide *a* (301).

As with Chl, completion of the BChl biosynthesis pathway is followed by transfer of the final product to the membrane-embedded machinery for photosystem assembly. In *R. sphaeroides*, BChl synthase associates with the protein translocase subunit YajC and the YidC membrane protein insertase and the assembly factor for the light-harvesting 1 complex, LhaA (302). It was proposed that membrane nanodomains foster interactions between pigments produced by BchG and nascent proteins within the SecYEG-SecDF-YajC-YidC assembly machinery to coordinate assembly of this light harvesting complex.

BChl *b* has the most red-shifted absorbance known among photosynthetic organisms, and the monomer in diethyl ether absorbs at 795 nm. This shift, relative to BChl *a* absorbing maximally at ∼771 nm, arises from the influence exerted by the C8 ethylidene group on the π-electron system of the macrocycle (281). Remarkably, incorporation of BChl *b* within the light-harvesting LH1 complex of *Blastochloris viridis* imparts a much larger bathochromic shift to 1023 nm, the basis of which was revealed by the 2.9 Å structure of the RC-LH1 complex (303). This red shift is much larger than that seen for BChl *a* bound within the light-harvesting 1 complex of *R. sphaeroides*, which absorbs maximally at ∼875 nm (304).

Tsukatani *et al.* (281) overexpressed *bchXYZ* of *B. viridis* in *E. coli* and showed that the recombinant proteins converted the 8-vinyl group of 8-vinyl Chlide *a* to a C8-ethylidene moiety, yielding BChlide *g*. Subsequent action by C3^1^ hydratase (BchF) and dehydrogenase (BchC) enzymes convert the C3 vinyl side chain to an acetyl group, producing BChlide *b*, which would become BChl *b* upon esterification of the C17 propionate by phytol (Fig. 11). Thus, the activity of the COR (BchXYZ) from *B. viridis*, which catalyzes the formation of the C8-ethylidene group, is distinct from the COR (BchXYZ) of *R. sphaeroides* that also possesses 8-vinyl reductase activity (281). Each of these auxiliary functions augments the central Chlide *a* oxidoreductase activity. Starting from an engineered strain of *R. sphaeroides* that produces BChl *b*, inactivation of the *bchF* gene and replacement of the native *bchG* gene with *bchG* from *Heliobacterium modesticaldum* resulted in a strain that produced BChl *g*_F_, BChl *g* esterified with farnesol (Fig. 11), again demonstrating that enzymes and reactions leading to BChls are now largely understood (305).

A few puzzles still remain to be elucidated, however. The reaction centers of *H. modesticaldum* contain four molecules of 8^1^-hydroxy-Chl *a* (306), and whereas this Chl *a* derivative can be produced spontaneously when BChl *g*_F_ is exposed to oxygen (307), it seems highly unlikely that this occurs *in vivo* because this bacterium is a strict anaerobe and is rapidly killed in the presence of oxygen. A second unresolved issue concerns the stereochemistry of the methylcarboxyl moiety at C13^2^. Bulk Chls and BChls have *R*-stereochemistry at C13^2^, but small amounts of pigments with *S*-stereochemistry at this position occur in reaction centers. Heterodimeric type-1 reaction centers, such as photosystem I, have a heterodimer of Chl *a*_P_ (*R*) and Chl *a*_P_′ (*S*) as the special pair P700, whereas homodimeric type-1 reaction centers have special pairs that are either BChl *a*_P_′, BChl *g*_F_′, or Zn-BChl *a*_P_′ (241, 308). How these C13^2^ epimers of BChls *a*_P_, BChl *g*_F_, and Chl *a*_P_ are produced is unknown. Finally, *Acidiphilium rubrum* exclusively produces Zn-BChl *a* (309), and the reaction centers of *Chloroacidobacterium thermophilum* have a Zn-BChl *a*_p_′ dimer as the special pair (308, 310, 311). It is unknown how Zn^2+^ replaces Mg^2+^ in either case, but this is especially perplexing in the latter case, because *C. thermophilum* mostly synthesizes BChl *a* and Chl *a* containing Mg^2+^ ions.

## *Chlorobium* Chls: BChls *c*, *d*, *e*, and *f*

**Figure 12.**
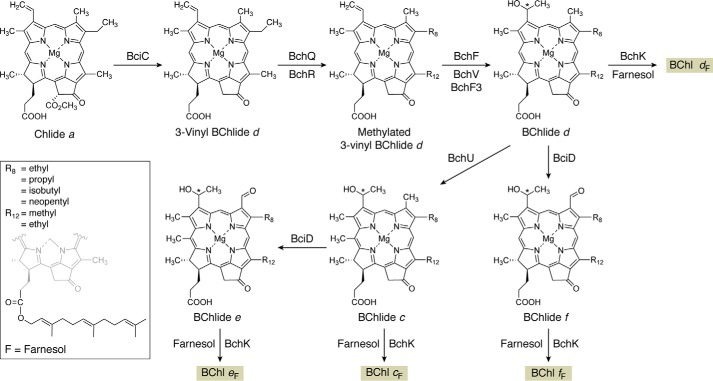
**Synthesis of bacteriochlorophylls *c, d, e*, and *f* from chlorophyllide *a*.** Purified BciD is active with both BChlide *d* and BChlide *c* as substrates. The conversion of the C7 methyl group to a formyl group proceeds via a geminal diol intermediate that spontaneously dehydrates to produce the formyl group. Esterifying alcohols are added by BchK in all cases. BChls *c* and *d* are characteristically found in green-colored green bacteria, whereas BChls *e* and *f* are produced by brown-colored green sulfur bacteria. Note that BChl *f* has not been observed in nature; however, it has been generated by mutation of *bchU* in *C. limnaeum* and can still produce functional chlorosomes (see section “*Chlorobium* Chls: BChls *c*, *d*, *e*, and *f*” for more details). In green sulfur bacteria, the esterifying alcohols of BChls *c, d, e*, and *f* are usually farnesol (*F*) (see *inset*, showing a farnesol group attached to ring D of a partial macrocycle). However, in members of the Chloroflexi and *C. thermophilum*, the esterifying alcohols are often highly variable and are frequently straight-chain alcohols or geranylgeraniol and its reduction products. All alcohols are added by BchK enzymes that are specific to individual organisms. In green sulfur bacteria and some other green bacteria, the ethyl side chain at C8 can be methylated by BchQ to produce propyl, isobutyl, and neopentyl side chains (see *inset*). Similarly, the methyl group at C12 can be methylated by BchR to produce an ethyl side chain. The *asterisk* near the hydroxyl group at C3^1^ indicates that this is a chiral center that is mostly *R* but is *S* when the side chains at C8 and C12 are more extensively methylated. The *shaded boxes* surrounding the names of some compounds coordinate with other pathway figures and the summary in Fig. 14.

Green bacteria are a highly diverse ensemble of bacteria from three phyla, Chlorobi, Chloroflexi, and Acidobacteria, but they share two interrelated and defining properties. All green bacteria employ chlorosomes as the light-harvesting antenna for phototrophic growth, and each green bacterium can synthesize one member of the so-called “*Chlorobium* Chls” (*i.e.* BChl *c*, *d*, *e*, or *f*) (222, 312, 313). These molecules are not actually BChls but are in fact chlorins, and, compared with Chl *a*, they have two important modifications. They have a hydroxyl group at the chiral C3^1^ position, and they lack the methylcarboxyl group at C13^2^ (Fig. 12). These two modifications allow these BChls to form protein-free, supramolecular aggregates in chlorosomes (314), the structures of which are now known (315–318). These supramolecular aggregates have emergent absorption properties that are not observed for the monomeric BChls in organic solvents. For example, BChl *c* has an absorbance spectrum that is very similar to that of Chl *a* in methanol with a maximum at approximately 663 nm, but BChl *c* in chlorosomes absorbs maximally at about 740–750 nm (312, 313). This large bathochromic shift allows organisms with BChl *c* to harvest light efficiently even if they are growing beneath a layer of organisms containing Chl *a*. Correspondingly, BChls *e* and *f* produce emergent, strong absorption bands between 500 and 550 nm in the blue-green region of the visible spectrum, which allows these organisms to grow at much greater depths in aquatic environments (242, 319).

The determination of the genome sequence for the naturally transformable GSB *C. tepidum* in 2002 provided an initial opportunity to search for the genes for BChl *c* biosynthesis (320, 321). *C. tepidum* naturally synthesizes three Chls/BChls: BChl *c*_F_ esterified with farnesol, Chl *a*_PD_ esterified with Δ2,6-phytadienol, and BChl *a*_P_ esterified with phytol. The *C. tepidum* genome encoded three Chl/BChl synthases (ChlG, BchG, and BchK), and deletion of *bchK* importantly showed that this gene encoded the BChl *c* synthase and that cells lacking BChl *c* were viable (322). These findings made it possible to search for the other genes required for BChl *c* biosynthesis by bioinformatics, neighborhood analysis, and reverse genetics approaches, which led to the elucidation of the entire pathway to these BChls (see Ref. 222 for a detailed review).

The first committed step in synthesis of BChls *c*, *d*, *e*, and *f* is removal of the C13^2^ methylcarboxyl group and branches from the hub compound Chlide *a* (Fig. 12). The *bciC* gene that encodes the demethoxycarbonylase enzyme was identified by phylogenetic profiling, mutagenesis of the corresponding gene in *C. tepidum*, and biochemical characterization of the mutant (323). The mutant was unable to synthesize BChl *c* and accumulated excess Chlide *a*. More recently, *bciC* was successfully expressed in *E. coli*, and the enzyme was shown to be active with Chlide *a*, 8-vinyl Chlide *a*, and some other substrates but exhibited a preference for Chlide *a* (324, 325). No activity was observed with porphyrin derivatives or when the C13^2^ methylcarbonyl group had *S*-stereochemistry, and the reaction was not inhibited by methanol, suggesting that the enzyme is a demethoxycarbonylase and not a methylesterase. These results demonstrate that BciC catalyzes the formation of 3-vinyl-BChlide *d* from Chlide *a* as the first committed step in the pathway to BChl *d* and BChl *c*.

The next reactions in the pathway may not occur in a specific order. Because methylation at the C8^2^ and C12^1^ positions affects the chirality of the product of the C3 vinyl group hydration reaction, it seems likely that these two methylation steps can precede hydration. However, in some strains (*e.g. Chloroflexus* sp.) and in *bchQ* and *bchR* mutant strains, these reactions do not occur at all, and the downstream reactions still occur. For example, hydration of the C3 vinyl group occurs (with mostly *R*-stereochemistry) in *bchQ* and *bchR* mutants, so clearly the C3-vinyl hydratase enzyme, BchF, does not require methylated substrates for its activity (see BChl *a* discussion above). The C8^2^ (BchQ) and C12^1^ (BchR) methyltransferase enzymes are members of the radical SAM methyltransferase superfamily (222, 326). BchR can methylate the C12 methyl group to produce an ethyl side chain at this position. BchQ can perform up to three methylations of the terminal carbon atom of the C8 ethyl group of 3-vinyl-BChlide *d*, producing compounds with *n*-propyl, isobutyl, or neopentyl side chains at C8. These methylations fine-tune the absorbance maximum of the BChl aggregates in chlorosomes and additionally affect the half-bandwidth of the emergent far-red absorbance maximum of the chlorosomes. One can rationalize these methylation reactions as the equivalent of changing the protein environment of chromophores in other light-harvesting antenna systems. The packing of the BChl aggregates in chlorosomes is affected locally by changes in methylation, and this produces inhomogeneous broadening of the near-IR absorbance band, which increases the probability of photon capture by the antenna pigments over a broader range of wavelengths (see Refs. 326 and 327 for further details).

The next step in this pathway is catalyzed by C3^1^ vinyl hydratase, BchF, which is shared with the pathway to BChl *a* in these organisms and produces *R*-stereochemistry at the chiral carbon, or BchV, which preferentially uses substrates with multiple methyl groups and preferentially produces *S*-stereochemistry at the chiral carbon (Fig. 12) (222, 328). A *bchF* mutant of *C. tepidum* makes significantly less BChl *a*, increased levels of 3-vinyl-BChl *a*, and less BChl *c* than the WT (329). The BChl *c* homologs in this mutant are primarily *S*-epimers, and compared with WT, the BChl *c* homologs in the mutant were more highly methylated at the C8^2^ position. A *bchV* mutant synthesized less BChl *c* than the WT, and most of the BChl *c* had *R*-stereochemistry at C3^1^. The amounts of ethyl and propyl side chains at C8 were similar to WT, but almost no BChl *c* with an isobutyl side chain at C8 was observed. Studies with recombinant BchF and BchV confirmed that these enzymes have C-3^1^ vinyl hydratase activity with a broad range of substrates (330, 331). Brown-colored GSB that synthesize BChl *e* have a third C3^1^ vinyl hydratase member of this family, but the function of that enzyme is unknown at present. It is not entirely clear why GSB have two or even three isoenzymes for this C3^1^ vinyl hydratase activity; nor is it clear what the value of producing BChls with different stereochemistry at C3^1^ might be.

Brown-colored GSB synthesize BChl *e*, and unlike BChls *c* and *d*, this pigment forms supramolecular aggregates in chlorosomes that have greatly enhanced absorption in the blue-green region of the visible spectrum. Because of this, these organisms characteristically occur at greater depths in aquatic environments, the most extreme example being a stable population of GSB that occurs 110 meters below the surface of the Black Sea (332, 333). Bioinformatic comparisons of green- and brown-colored strains identified a small cluster of genes uniquely found in BChl *e*–producing organisms (334–336). One of the genes in this cluster, *bciD*, encoded a member of the radical SAM superfamily. Deletion of the *bciD* gene in the model, brown-colored GSB, *Chlorobaculum limnaeum*, showed that this gene was necessary for BChl *e* biosynthesis (336, 337). Studies with recombinant, reconstituted BciD showed that this radical SAM enzyme is sufficient for BChl *e* biosynthesis; it catalyzes two successive hydroxylations of the C7 methyl group of BChlide *c* or BChlide *d* to yield BChlide *e* or BChlide *f*, respectively (336). The resulting *geminal* diol intermediates presumably lose water spontaneously to produce the C-7 formyl groups of BChl *e* or BChl *f*, respectively (Fig. 12).

BChl *c* and BChl *e* have a methyl group at C20, which is introduced by BchU, the C20 methyltransferase. Inactivation of the *bchU* gene in *C. tepidum* produces a strain that synthesizes BChl *d*, and a strain that naturally produces BChl *d* was further shown to carry a frameshift mutation in the *bchU* gene (327). The crystal structure of dimeric BchU has been determined (338), and the C3^1^ hydroxyl group appears to be an important determinant of substrate binding, suggesting that C20 methylation follows hydration of the C3 vinyl group. Growth competition studies with FRL show that cells producing BChl *c* have a growth advantage over otherwise identical cells producing BChl *d*. Mutation of the *bchU* gene in *C. limnaeum*, which normally produces BChl *e*, yielded a mutant with chlorosomes containing BChl *f*, an anticipated pigment that has surprisingly never been observed naturally (242, 339). The mutants grew more slowly under low light intensity than the WT, probably because energy transfer from BChl *f* to BChl *a* in the chlorosome baseplate was less efficient than for BChl *e* to BChl *a* transfer (319, 340). The triplet excitonic state of BChl *f* is more favorable energetically for the production of singlet oxygen than the triplet excitonic states of BChls *c*, *d*, and *e*, and this difference could lead to excessive oxidative stress when cells producing BChl *f* are exposed to high light. The energy levels of the triplet excitonic states of the naturally occurring BChls *c*, *d*, and *e* may provide some natural protection against the production of singlet oxygen and associated oxidative stress in green bacteria (341). For all of these reasons, BChl *f* seems not to occur naturally, making it a “forbidden” BChl (242, 319, 339–341).

The branching pathways for the synthesis of these four BChls terminate like those of all other Chls and BChls with a Chl/BChl synthase that adds the esterifying alcohol moiety to the tetrapyrrole headgroup (Fig. 12). As noted above, biochemical studies have shown that (B)Chl synthases exhibit strong substrate preference for the tetrapyrrole but much lower specificity for the esterifying alcohol (342, 343). The genome of *C. tepidum* and other green-colored GSB encode three (B)Chl synthases (320), and phylogenetic analyses indicated that the *bchG* (CT1610) and *chlG* (CT1270) genes were likely to encode the BChl *a* synthase and Chl *a* synthase, respectively (322). The *bchG* genes of *C. tepidum* and *Chloroflexus aurantiacus* were identified by functional complementation of a *bchG* mutant of the purple bacterium *R. capsulatus* (344), which confirmed that ORF CT1610 of the *C. tepidum* genome was BChl *a* synthase. Heterologously produced BchG showed strong substrate specificity for BChlide *a* and was unable to esterify BChlide *c* or Chlide *a* (343). However, when farnesyl-pyrophosphate was provided as the other substrate instead of geranylgeranyl-pyrophosphate, BchG readily catalyzed the formation of BChl *a*_F_. No *in vitro* or *in vivo* complementation experiments for the *chlG* gene of GSB have yet been described. However, characterization of *bchP* and *bciC* mutants of *C. tepidum* led to the inference that ChlG can use geranylgeranyl diphosphate as a substrate (222).

Inactivation of the third synthase gene, *bchK*, produced a mutant that could no longer synthesize BChl *c*_F_, thus establishing clearly that BchK is BChl *c* synthase that adds the farnesyl tail to BChlide *c* (322). Because *bchU* mutants of *C. tepidum* readily synthesize BChl *d*_F_, BchK must additionally be able to utilize BChlide *d* as a substrate (327). The *bchK* mutant is unable to synthesize BChl *c*, so it is likewise unable to produce functional chlorosomes and instead produces vestigial chlorosomes filled with carotenoids (“carotenosomes”) (322). Although it had not yet been named at the time, BChl *c* synthase activity was demonstrated with a BchG paralog from *C. aurantiacus* (343). Some GSB produce a paralog of BchK, denoted as BchK2 (222, 336), and interestingly, all BChl *e*–producing GSB produce at least one BchK2 paralog. At present, the roles of these paralogous BChl *c* synthases are unknown. Feeding studies with *C. tepidum* provided with a wide variety of exogenous alcohols suggest that the substrate specificity of BchK for the esterifying alcohol is extremely broad and is primarily driven by substrate availability (342, 345–349).

Sequential reduction of geranylgeraniol as the free pyrophosphate or as the alcohol after attachment to a tetrapyrrole headgroup leads to Δ2,6-phytadienol or phytol, the esterifying alcohols of Chl *a*_PD_ and BChl *a*_P_, respectively. BChl *a*–producing members of the phylum Chloroflexi and the acidobacterium *C. thermophilum* have single copies of *bchP*, which thus must be responsible for the synthesis of phytol or both phytol and Δ2,6-phytadienol, respectively (222). However, *C. tepidum* has two *bchP* paralogs, and a few GSB have more than two. A null mutant of one of these, denoted *bchP*, showed that the product of this gene is required for geranylgeranyl reductase activity (293, 350). More recent studies have shown that the second paralog, formerly *bchO* and now renamed *cruI* to reflect its role in carotenoid biosynthesis, reduces the 1,2-double bond of chlorobactene (351). In organisms with other *bchP* paralogs, the substrates are unknown.

## Bilins: Chromophores of phycobiliproteins, phytochromes, and cyanobacteriochromes

Bilins are linear tetrapyrroles derived from heme, are found in all domains of life, and perform a wide range of functions. Biliverdin IXα is a green-colored bilin that is the primary product of heme catabolism. It is derived from the dioxygen-dependent cleavage of the heme macrocycle of hemoglobin of erythrocytes in animals by a reaction catalyzed by heme oxygenase. In humans, biliverdin IXα is usually rapidly reduced by NADPH-dependent biliverdin reductase to produce the yellowish tetrapyrrole, bilirubin (352). Both compounds have antimutagenic and antioxidant properties, and both are potent scavengers of peroxyl radicals (353, 354). Further reduction of bilirubin in the colon leads to the production of stercobilin, a compound responsible for the brown color of human feces. In fungi and bacteria, biliverdin IXα is the light-responsive chromophore of phytochrome photoreceptors (20, 355).

Heme oxygenases occur in all three domains of life, but at least five families of heme-degrading enzymes have now been described (15, 16, 20, 355–357). The “canonical” heme oxygenase, HO-1, of eukaryotes and some aerobic bacteria cleaves heme releasing biliverdin IXα, ferrous iron, and carbon monoxide (15, 16). The enzyme catalyzes cleavage of the heme ring at the α-*meso* bridging carbon to form biliverdin IXα in a reaction that proceeds by three successive monooxygenase-like reactions with α-*meso*-hydroxyheme and verdoheme intermediates. In humans, much of the heme oxygenase activity is found in the spleen, which can degrade up to ∼1% of erythrocyte heme per day, accounting for about 80% of the daily production of carbon monoxide.

An extremely important virulence determinant among human pathogens is the capacity to obtain Fe when living inside the host. Iron (and heme) trafficking is very tightly controlled and regulated in animals, and hemoglobin is an abundant source of both heme and iron under these growth conditions. To release Fe, however, pathogens must cleave the heme macrocycle. Whereas it has long been known that some pathogens use Hox1 or Hox2 to perform this task, it has recently been discovered that other types of heme oxygenases occur among bacteria (and eukaryotes). A second major family of heme oxygenases, the IsdG family, comprises proteins that are structurally distinct from HO-1 and related enzymes, and whereas HO-1 enzymes produce biliverdin and carbon monoxide as products, IsdG family members produce several different bilin products and only sometimes release carbon monoxide. IsdG family members have been identified in all three domains of life, and several examples have been characterized. *Staphylococcus aureus* produces two members of the IsdG family, IsdG and IsdI, which produce formaldehyde and “staphylobilin,” a mixture of 5-oxo-δ-bilirubin and 5-oxo-β-bilirubin (358). The MhuD enzyme of *Mycobacterium tuberculosis* produces mycobilin, which is a derivative of 5-oxo-β-bilirubin in which the bridging carbon is retained as a formyl group (359–361). The green alga, *C. reinhardtii*, can produce two members of the HO-1 family and one member of the IsdG family. The bilin product of the latter enzyme has not yet been structurally characterized, but it is known to be distinct from biliverdin and the products of other IsdG-type enzymes (362). Recently, ChuW, a novel oxygen-independent, heme-degrading enzyme from *Escherichia coli* O157:H7, has been described. This enzyme is a member of the radical SAM methyltransferase family that produces a linear tetrapyrrole, anaerobilin, by cleavage of heme in the absence of dioxygen (356). Finally, there are possibly two other families of heme-degrading enzymes, but key details of the reactions these enzymes catalyze and the products they form are still unknown (357).

**Figure 13.**
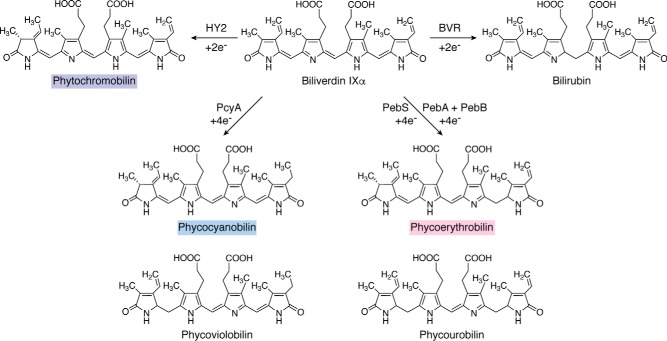
**Synthesis of bilins from biliverdin, which is formed from heme *b*.** Biliverdin is produced by the oxygen-dependent cleavage of heme *b* by the enzyme heme oxygenase. Regio-specific ferredoxin-dependent bilin reductases lead to phytochromobilin, phycocyanobilin, and phycoerythrobilin, and biliverdin reductase produces bilirubin. Note that phycoerythrobilin can be synthesized by one enzyme (PebS) or in two steps by PebA and PebB via a 17,18-dihydrobiliverdin intermediate. Phycoviolobilin and phycourobilin are produced from phycocyanobilin and phycoerythrobilin, respectively, by isomerization, which occurs during the attachment of these bilins to proteins by isomerizing bilin lyases. *BVR*, biliverdin reductase; *HY2*, phytochromobilin synthase. See section “Bilins: Chromophores of phycobiliproteins, phytochromes, and cyanobacteriochromes” for additional details. The *shaded boxes* surrounding the names of some compounds coordinate with the summary in Fig. 14.

Phycobiliproteins are brilliantly colored, water-soluble, light-harvesting proteins found in cyanobacteria and in red algae, glaucophytes, and cryptomonads. The subunits of these proteins carry one or more bilin chromophores, which are bound to the proteins via thioether linkages to cysteine residues. Phycocyanobilin and phycoerythrobilin are the most common chromophores (16, 363). Cyanobacteria typically have two forms of heme oxygenase, one of which is produced constitutively (Hox1) and another that is synthesized under microoxic conditions (Hox2) (364). In plants, algae, and cyanobacteria, various bilins are produced from biliverdin by enzymes known as ferredoxin-dependent bilin reductases (FDBRs) (for a review, see Ref. 17). For example, phycocyanobilin is produced from biliverdin IXα by PcyA (Fig. 13), which carries out two successive regio-specific reductions by a radical mechanism (17, 365). Phycoerythrobilin, which is an isomer of phycocyanobilin, can be produced by three different enzymes (17). PebA and PebB are members of the FDBR family of enzymes and can catalyze successive reductions of biliverdin to produce phycoerythrobilin via a 15,16-dihydrobiliverdin intermediate (366). Alternative FDBR family members, PebS (367) and PcyX (368), were found in marine viral genomes and can catalyze two reduction steps via a different intermediate than PebA/PebB to produce phycoerythrobilin (Fig. 13) (18).

In addition to their important roles in light-harvesting proteins, biliverdin, phytochromobilin, phycocyanobilin, and phycoviolobilin can be covalently bound to GAF domains of phytochromes and phytochrome-like cyanobacteriochrome photoreceptors (19). GAF domains (cGMP-specific phosphodiesterases, adenylyl cyclases, and FhlA) occur in diverse proteins and are structurally related to Per-Arnt-Sim (PAS) domains, both of which frequently occur in sensory proteins. Cyanobacteria do not naturally synthesize phytochromobilin, but this chromophore binds to GAF domains of plant phytochrome photoreceptors and is photoactive. Phycoviolobilin is produced by isomerization of phycocyanobilin after covalent attachment to the Cys residue in the GAF domain. Several other mechanisms, including protonation of the bilin, cysteine adduct formation to the central bridging carbon of the chromophore, and steric hindrance, can all be used to further tune the wavelength response of cyanobacteriochromes and phytochromes (19). Thus, members of this family of photoreceptors can sense and respond to light wavelengths between 350 and 750 nm.

Attachment of bilins to phycobiliprotein subunits usually requires specific lyases, some of which are also bilin isomerases (369). For example, the phycobiliprotein phycocyanin carries one phycocyanobilin on its α subunit and two phycocyanobilins on its β subunit (365). The α subunit chromophore is attached by a member of the CpcE/CpcF family of bilin lyases (370–372). The β subunit chromophore at Cys-82 is attached by a member of the CpcS (or CpcS/CpcU) family (373, 374), and the chromophore at Cys-153 of the β subunit is attached by CpcT (375). Phycobiliviolin and phycourobilin also occur in cyanobacteria, but to date, no FDBR members have been identified that can directly synthesize these two chromophores in cyanobacteria. Instead, some members of the CpcE/CpcF family isomerize these bilin chromophores during attachment to the apoprotein. For example, the PecE/PecF lyase for the α subunit of phycoerythrocyanin isomerizes phycocyanobilin to phycobiliviolin during attachment of phycocyanobilin to PecA, and other lyases can isomerize phycoerythrobilin to phycourobilin during the attachment reactions with linker proteins or phycoerythrin subunits (376–378). Interestingly, the moss, *Physcomitrella patens*, has an FDBR, PubS, that can synthesize phycourobilin directly from biliverdin (379). Phycourobilin does not appear to be used for light harvesting but instead seems to be involved in light sensing and photomorphogenesis. The last member of the FDBR family of enzymes is phytochromobilin synthase, HY-2, which produces the phytochromobilin chromophore of plant phytochromes (380). Surprisingly, when produced in *E. coli*, phytochromobilin can be attached to the α subunit of apophycocyanin from *Synechocystis* sp. PCC 6803. Although this protein only binds phycocyanobilin naturally, when co-expressed along with various bilins in *E. coli*, it can carry any one of six different bilin chromophores produced by means of the enzymes described above (381). These variants have potential biotechnological applications.

## Conclusions

**Figure 14.**
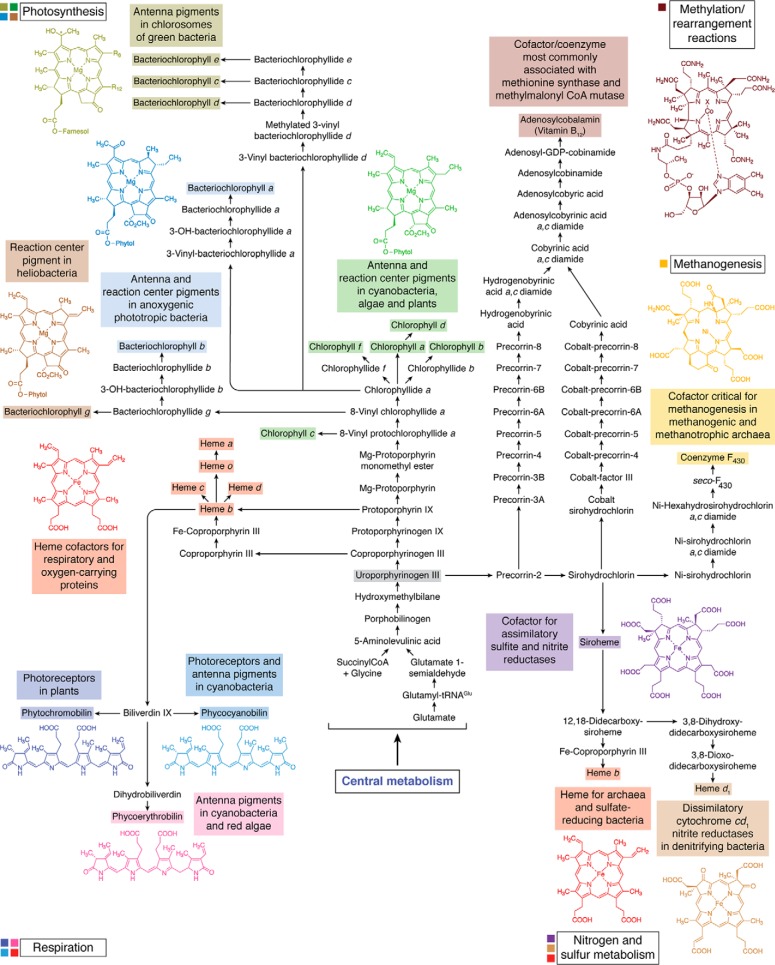
**Summary overview of the entire tetrapyrrole biosynthetic network.** Those aspects of life on Earth that depend on tetrapyrroles are shown at the periphery in *boxes* with *black outlines*, adjacent to *color-coded squares* that correspond to various tetrapyrroles. *Color-coded boxes* specify the functions of nearby tetrapyrroles. The *colors* used in this figure coordinate with the *colored boxes* associated with the names of end-product compounds in other figures in this article. The link to central metabolism is indicated, which provides the starting point for the entire network. Note that all compounds produced in this pathway ultimately are derived from uroporphyrinogen III. Note also that heme *b* arises as a respiratory cofactor and as a precursor of hemes *c*, *d*, and *o* and biliverdin IX. Heme *b* also arises separately in archaea and sulfate-reducing bacteria as a cofactor for nitrogen and sulfur metabolism.

This review summarizes and integrates the ∼90 biosynthetic steps that, starting with the simple C5 precursor ALA, culminate in the production of all of the major tetrapyrrole cofactors required for life on Earth. These tetrapyrrole cofactors underpin respiration, nitrogen and sulfur metabolism, mammalian metabolism, methanogenesis, and photosynthesis, and a scheme depicting the complete network of tetrapyrrole biosynthetic reactions is shown in Fig. 14.

Given the fundamental importance of the tetrapyrrole macrocycle for the functional roles of hemes, Chls, vitamin B_12_, and coenzyme F_430_, it is significant that this vital framework is established almost at the outset, with just three steps required to form uroporphyrinogen III from ALA. Uroporphyrinogen III is the precursor for vitamin B_12_ and coenzyme F_430_, and only two more steps are required to form the Copro III and Proto IX precursors of other hemes and bilins and of the myriad Chl and BChl pigments that collect the solar energy that powers much of the biosphere. The intermediates and final products in the Chl and BChl branches in Fig. 14 are notable for their light-absorbing properties, and as one moves along each branch of these pathways, there is a progression in their abilities to selectively absorb certain parts of the solar spectrum. The Granick hypothesis, proposed over 70 years ago, suggests that pathways evolve as organisms evolve (and conversely, organisms evolve as pathways evolve; also sometimes stated as the biosynthetic pathway of Chl recapitulating its evolution) (382–384). Thus, the proliferation of Chl and BChl branches in Fig. 14 is proposed to reflect the acquisition of new biosynthetic steps and the production of novel pigments, allowing phototrophs to avoid competition by seeking and occupying new spectral niches. Equally importantly, biosynthetic alternatives were developed in response to dioxygen in the atmosphere, leading to parallel pathways for nearly all of these essential molecules (5, 223, 385, 386). In this respect, there are parallels with the anaerobic and aerobic pathways for biosynthesis of vitamin B_12_. Although the Granick hypothesis was formulated for hemes and Chls, this postulate could be relevant to all of the biosynthetic reactions in Fig. 14.

Although there are still many details to unravel, the biosynthetic framework of tetrapyrrole biosynthesis is now reasonably well-understood. However, the regulation of these pathways and their multiple branches; the stoichiometry, cellular location, and organization of pathway enzymes into biosynthetic complexes and assemblies; and the way in which the final cofactor products are incorporated within their respective protein scaffolds are largely unknown. Undoubtedly, many of these details will emerge in the decades to come.

## Footnotes

5 G. Shen and D. A. Bryant, unpublished observations.

6 A. Hitchcock, personal communication.

## Footnotes

4 The abbreviations used are:

Chl: chlorophyll
BChl: bacteriochlorophyll
ALA: aminolevulinic acid
GSA: glutamate semialdehyde
PBG: porphobilinogen
HMB: hydroxymethylbilane
ProtoIX: protoporphyrin IXα
Chlide: chlorophyllide
PChlide: protochlorophyllide
BChlide: bacteriochlorophyllide
LPOR: NADPH:protochlorophyllide oxidoreductase
DPOR: dark-operative protochlorophyllide reductase
GSB: green sulfur bacteria
CAO: Chl *a* oxygenase
FRL: far-red light
COR: Chlide *a* oxidoreductase
3HE: 3-hydroxyethyl
FDBR: ferredoxin-dependent bilin reductase.
